# Differential Radiomics‐Based Signature Predicts Lung Cancer Risk Accounting for Imaging Parameters in NLST Cohort

**DOI:** 10.1002/cam4.70359

**Published:** 2024-10-28

**Authors:** Leyla Ebrahimpour, Philippe Després, Venkata S. K. Manem

**Affiliations:** ^1^ Centre de Recherche du CHU de Québec Université Laval Canada; ^2^ Department of Radiology and Imaging Sciences Emory University Atlanta Georgia USA; ^3^ Département de Physique, de Génie Physique et D'optique Université Laval Canada; ^4^ Centre de Recherche de l'Institut Universitaire de Cardiologie et de Pneumologie de Québec Canada; ^5^ Big Data Research Center Université Laval Canada; ^6^ Cancer Research Center Université Laval Canada; ^7^ Département de Biologie Moléculaire, Biochimie Médicale et Pathologie Université Laval Canada

**Keywords:** ComBat harmonization, delta radiomic features, lung cancer, machine learning, radiomics

## Abstract

**Objective:**

Lung cancer remains the leading cause of cancer‐related mortality worldwide, with most cases diagnosed at advanced stages. Hence, there is a need to develop effective predictive models for early detection. This study aims to investigate the impact of imaging parameters and delta radiomic features from temporal scans on lung cancer risk prediction.

**Methods:**

Using the National Lung Screening Trial (NLST) within a nested case–control study involving 462 positive screenings, radiomic features were extracted from temporal computed tomography (CT) scans and harmonized with ComBat method to adjust variations in slice thickness category (TC) and reconstruction kernel type (KT). Both harmonized and non‐harmonized features from baseline (T0), delta features between T0 and a year later (T1), and combined T0 and delta features were utilized for the analysis. Feature reduction was done using LASSO, followed by five feature selection (FS) methods and nine machine learning (ML) models, evaluated with 5‐fold cross‐validation repeated 10 times. Synthetic Minority Oversampling Technique (SMOTE) was applied to address class imbalances for lung cancer risk prediction.

**Results:**

Models using delta features outperformed baseline features, with SMOTE consistently boosting performance when using combination of baseline and delta features. TC‐based harmonized features improved performance with SMOTE, but overall, harmonization did not significantly enhance the model performance. The highest test score of 0.76 was achieved in three scenarios: delta features with a Gradient Boosting (GB) model (TC‐based harmonization and MultiSurf FS); and T0 + delta features, with both a Support Vector Classifier (SVC) model (KT‐based harmonization and *F*‐test FS), and an XGBoost (XGB) model (TC‐based harmonization and Mutual Information (MI) FS), all using SMOTE.

**Conclusions:**

This study underscores the significance of delta radiomic features and balanced datasets to improve lung cancer prediction. While our findings are based on a subsample of NLST data, they provide a valuable foundation for further exploration. Further research is needed to assess the impact of harmonization on imaging‐derived models. Future investigations should explore advanced harmonization techniques and additional imaging parameters to develop robust radiomics‐based biomarkers of lung cancer risk.

## 1 Introduction

Cancer is one of the three leading causes of death among individuals aged 30–69 in 177 out of 183 countries [1]. Lung cancer remains the leading cause of cancer‐related mortality worldwide, with early detection being critical for improving survival rates [2]. According to the Canadian Cancer Statistics of the Canadian Cancer Society in 2023, lung cancer remains the most commonly diagnosed cancer in Canada with an estimated 1 in 14 Canadians (7%) expected to be diagnosed in their lifetime. Most patients with lung cancers are diagnosed at advanced stages, which explains the poor prognosis of this disease even with modern therapeutic interventions such as immunotherapy. Reducing lung cancer mortality depends on two main strategies: identifying and mitigating risk factors, and early detection through screening [3]. The imaging technology, especially low‐dose computed tomography (LDCT), has significantly improved the early detection and monitoring of lung nodules. In the last few years, LDCT has emerged as an effective technique at identifying individuals that are at a higher risk of developing pulmonary cancers [4, 5, 6, 7]. In this regard, two NLST seminal studies have shown a decrease in lung cancer deaths when patients were screened with LDCT [6].

The National Lung Screening Trial (NLST) demonstrates the benefits of lung cancer screening conducted over a 3‐year period [6]. However, potential harms are associated with the implementation of a lung cancer screening program which include false‐positive results leading to unnecessary tests and invasive procedures. In the NLST cohort, the majority (96.4%) of positive LDCT screens were found to be false positives or intermediate pulmonary nodules (IPNs) [8]. The additional clinical resources (access to physicians, radiology, pathological procedures) to obtain a reasonable balance of benefits over harms represents a significant challenge in the context of a public health system. While there are clinical guidelines [9, 10] to assess and monitor nodules, the lack of validated tools to predict lung cancer risk remains a challenge. A precise and reliable non‐invasive tool is crucial to assist radiologists and pulmonologists in effectively managing nodules within lung cancer screening protocols. Recent advancements in computerized tools capable of converting images into quantitative data, known as radiomics followed by their analysis using artificial intelligence (AI) algorithms, have shown promise in addressing this need [11]. The data and images from the NLST cohort have proven to be an invaluable resource for numerous significant radiomic investigations [8, 12, 13, 14].

Radiomics, the extraction and analysis of large amounts of advanced quantitative imaging features from medical images like CT scans, has shown promise in building prognostic and diagnostic biomarkers of cancer [11, 15, 16, 17]. Radiomic features extracted from regions of interest (ROIs) characterize the attributes such as shape, texture, and the intensity of X‐ray absorption within the ROIs. Radiomics studies are typically conducted with images taken at a single time‐point; however, the temporal variations in radiomic features known as delta radiomics have shown potential for providing predictive insights [8, 18]. In addition, the variations in imaging protocols across institutions including the CT slice thickness and reconstruction approaches can lead to heterogeneous datasets which can significantly impact the predictive model development [19]. Moreover, slice thickness directly affects the quality of 3D models generated from volumetric data. Larger slice thicknesses lead to reduced spatial resolution in the longitudinal direction [20]. The reconstruction kernel, also known as the reconstruction filter, is another crucial CT scanner parameter that significantly influences the texture and spatial resolution of the resulting image. Hence, the choice of the reconstruction kernel can also lead to variations in quantitative analysis using radiomics irrelevant to underlying anatomical structures [21]. Because of these potential variations, it is essential to address the inconsistencies associated with these imaging parameters on the predictive models. The necessity and impact of harmonizing radiomic features extracted from medical images have been investigated in several studies [22, 23, 24, 25, 26]. However, few studies have employed harmonized radiomic features to develop predictive models and it remains unclear whether incorporating harmonization techniques will enhance the predictive performance of these models [25, 27, 28]. Regarding the use of harmonized radiomic features for lung cancer prediction using NLST data, we found a study where harmonization was performed only based on cohort differences used in the study [28].

In this study, we aimed to investigate the impact of imaging parameters and delta radiomic features on lung cancer risk prediction using a subsample of NLST data by utilizing radiomic features extracted from the longitudinal scans. We employed the ComBat harmonization method [29] to address the variations in slice thickness and reconstruction kernel, while comparing outcomes across various lung cancer risk predictive models. Additionally, we integrated a balancing method to address dataset imbalances, providing insights into different approaches for improving predictive models. *To the best of our knowledge, this is the first study within the NLST to explore the optimal lung cancer risk prediction model by employing a compendium of ML models and feature selection methods, considering harmonization, delta radiomic features, coupled with dataset balancing approaches*. This would enable us to identify appropriate feature selection and machine learning (ML) methods that can be used to develop clinically relevant and robust imaging‐based models for early detection of lung cancer.

## 2 Materials and Methods

### 2.1 Description of the Study Population

This study utilized a subset of anonymized LDCT images sourced from NLST [30, 31] which has been used as a nested case–control sample by Cherezov et al. [8] in an earlier study. The NLST is a controlled, multicenter trial that compares the effectiveness of LDCT scans and chest X‐rays to detect lung cancer in high‐risk individuals. Participants in the trial were current or former smokers, aged 55 to 77, with a smoking history of at least 30 pack‐years; and those who had quit had done so within the last 15 years. The NLST conducted three annual screenings: an initial baseline screen (T0) and two subsequent annual follow‐up screens (T1 and T2). According to the trial's criteria, a positive LDCT screen was defined by the detection of one or more non‐calcified nodules or masses exceeding 4 mm in axial diameter, or less frequently, other signs such as adenopathy or pleural effusion. Participants with positive screening results received follow‐up instructions according to the trial's guidelines. The cohort selected for this research excluded participants with only negative screening results, which were defined as scans showing no abnormalities, minor abnormalities not suggestive of lung cancer, or significant abnormalities deemed not suspicious for lung cancer.

### 2.2 Nested Case–Control Study Framework

**FIGURE 1:**
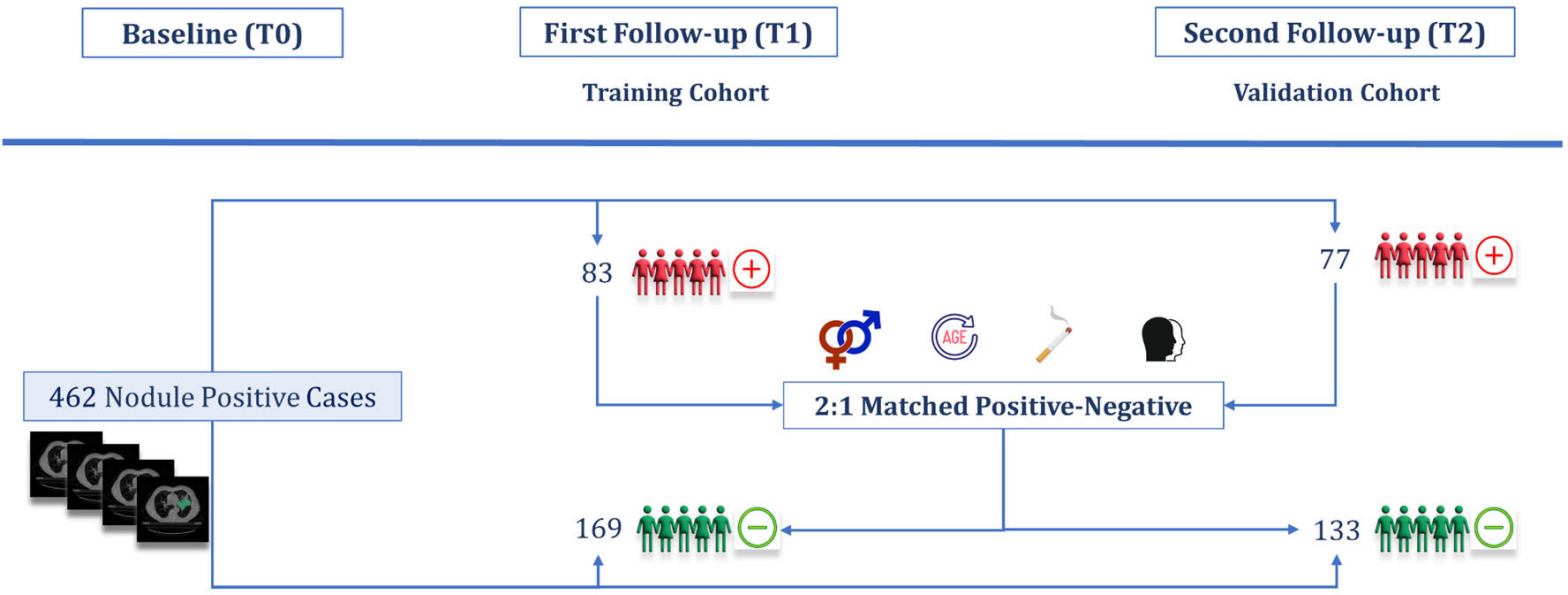
A schematic representation of the nested matched case–control study using the NLST dataset.

A schematic representation of the nested case–control study has been shown in Figure 1. In this study, we analyzed a total of 462 cases with positive initial screenings (T0). From this group, 160 participants were diagnosed with lung cancer during the first (T1) or second (T2) follow‐up screenings, labeled as positive cases. And, 302 participants were diagnosed as benign during all three screenings, labeled as negative cases. Based on these frameworks, two distinct cohorts were established. Cohort 1 consisted of positive cases diagnosed with lung cancer at T1, and Cohort 2 included those diagnosed at T2. The remaining negative cases, who had positive initial screenings but were not diagnosed with lung cancer from T0 through T2, were distributed between the two cohorts following a 2:1 nested case–control study design in a way that the age, sex, race, and smoking status distribution matched between negative and positive cases in both cohorts. This resulted in minimizing the influence of confounders between two groups, as described in earlier studies [8, 32]. This stratification of samples in a nested case–control framework resulted in 168 negative cases in Cohort 1 and 133 in Cohort 2. Ultimately, Cohort 1 served as the training dataset with 254 samples, while Cohort 2 was utilized as the validation dataset with 208 samples. This approach allowed us to use the baseline CT images to train and validate lung cancer risk prediction models for 1‐ and 2‐year intervals.

### 2.3 CT Annotation

Before the extraction of radiomic features from regions of interest, it was necessary to identify and segment lung nodules in both positively and negatively diagnosed cases in T0, T1, and T0 screening timelines. The nodule segmentations were obtained from the Schabath et al. study [32], which were performed manually by two radiologists, YL and QL, who reviewed all LDCT images. The process of identifying cancerous nodules among detected lung cancers during screenings utilized data from NLST, which provided details on nodule size and location. In instances where location data were incomplete, YL, the primary radiologist, manually identified and traced each nodule from T0 to T1. For participants with multiple nodules, the largest nodule identified at T0, and its changes through subsequent screenings were selected for the extraction of radiomic features [33].

### 2.4 Radiomic Feature Extraction and Preprocessing

For this analysis, we utilized PyRadiomics version 3.0.1, a comprehensive open‐source Python package for the extraction of detailed radiomic features from medical imaging data within specified regions of interest (ROI). These features were extracted from available CT images at T0, T1, and T2 screening timelines for Cohorts 1 and 2. The radiomic features can be categorized into four main groups [34, 35]:

- *Intensity‐based features:* These are first‐order statistical features that quantify characteristics of the tumor's intensity. Derived from the pixel intensity distribution within the ROI, these features include statistical measures like mean, median, mode, percentiles, skewness, kurtosis, and entropy. Each feature provides insights into the overall intensity distribution aiding in the differentiation of various tissue histologies. For example, mean intensity is calculated by averaging all pixel values, whereas entropy measures the randomness of the intensity distribution.
- *Shape‐based features:* These features can be calculated in both two and three dimensions providing insights into the geometric characteristics of the ROI. They include metrics such as volume, surface area, compactness, and sphericity. For example, volume is the sum of all voxel volumes within the ROI, and sphericity measures how closely the shape of the ROI approximates a sphere, providing information on the morphological properties of the tumor.
- *Texture features:* These features assess the spatial relationships and statistical dependencies between neighboring voxels. Techniques such as the gray‐level co‐occurrence matrix (GLCM) and gray‐level run length matrix (GLRLM) are used to derive these features. For instance, GLCM‐based contrast quantifies the local variations in pixel intensity, providing insight into the texture and pattern consistency within the ROI. These features are crucial for analyzing tissue heterogeneity and structural complexity.
- *Filter‐based features:* These features are derived from images that have been modified using specific filters, including Wavelet, Laplacian of Gaussian (LoG), Gradient, Square, Square‐root, and Exponential filters that will enrich the analysis of both texture and intensity characteristics. For instance, applying a Wavelet filter allows for the extraction of features that capture variations in pixel intensities across multiple scales and orientations, providing a more detailed interpretation of the imaging data.

Before the extraction of radiomic features, in‐plane resampling was performed using the B‐spline interpolation method from the SimpleITK (Simple Insightful Toolkit) library. This method adjusted the voxel size to (1,1, slice thickness) during preprocessing, which normalized the pixel spacing while maintaining the original slice thickness from the non‐resampled images. Normalizing is crucial for ensuring consistency, standardized representation, and proper voxel alignment. The choice to preserve the original slice thickness was strategic, aiming to later harmonize the extracted radiomic features based on batch parameters, including variations in slice thickness. Also, the bin width of 0.03 was used for the gray‐level discretization of the ROIs. Within the preprocessing step, the gray levels (voxel intensities) are resampled within the ROI to 2^n^ number of bins, where n varies from 3 to 8 in the literature [36, 37]. This approach not only reduces noise but also increases the stability of the features [38, 39]. The bin width of 0.03 was determined based on the range of the first‐order feature for the whole studies in this research, which was between 0.3 and 3 for the majority of the studies. This choice ensures that the number of bins remains between 10 and 100, a range that has demonstrated good reproducibility and performance in previous studies when using a fixed bin count [34, 40].

For the majority of studies, CT images were found for either T0 or T1 timelines with different reconstruction kernels used for image reconstruction. For each study, we selected CT images with the same reconstruction kernel at both T0 and T1 screenings. If multiple CT image pairs were found each with the same reconstruction kernel, one was chosen randomly, as there was no specific preference for the type of reconstruction kernel used.

### 2.5 Baseline and Delta Features

In this study, we analyzed six sets of radiomic features derived from CT scans taken at T0 and T1. For our analysis, we considered three main feature sets: the original features from T0, the delta features, and a combination of both, labeled as T0 + delta. Each of these sets was analyzed both before and after harmonization, resulting in six distinct sets of features used in this research. In addition, to explore changes in radiomic features over time, we also computed the difference between the features extracted at T0 and T1 time points, which is termed delta features in this work. This approach provided a comprehensive overview of the changes in nodules over time while controlling for potential confounding factors.

### 2.6 Harmonization of Features

In this study, we employed the Nested ComBat [19] method for harmonizing radiomic features based on two batch parameters, that is, slice thickness category (TC) and reconstruction kernel type (KT), independently. This step was adjusted for variations due to slice thickness and reconstruction kernels, ensuring standardized comparisons across all scans. Radiomic features extracted from CT images taken at T0, T1, and T2 were stratified into three slice thickness categories: 1.0 to 2.0 mm, 2.0 to 3.0 mm, and 3.0 to 4.0 mm. The reconstruction kernels used were also categorized into sharp and soft types. Sharp kernels included types such as “D,” “B70f,” “B80f,” “BONE,” “FC82,” “LUNG,” “B60f,” “FC51,” “FC30,” “FC50,” while soft kernels were identified as “B30f,” “B30s,” “FC10,” “FC02,” “FC01,” “A,” “STANDARD,” “B,” “C,” “B50s,” and “B50f.” Given that the CT images were produced by four different manufacturers, and considering that kernel implementation scan vary across these manufacturers, we aligned the kernels based on existing research that explores how reconstruction kernels influence radiomic features [41]. The clinical covariates used along with TC and KT, serving as batch effects, were smoking, gender, and age.

The Nested ComBat technique expands upon the traditional ComBat approach, which typically addresses a single batch effect, by accommodating multiple concurrent influences. Nested ComBat initiates the harmonization process with a list of predetermined batch effects and clinical covariates alongside the initial dataset of radiomic features. The process is iterative, that is, the features are adjusted individually for each batch effect in each cycle. This step is crucial for maintaining the clinical relevance of the features while ensuring that prior adjustments for other batch effects are not retained, thereby fostering a more focused and effective correction. After adjusting the features in each cycle, the Kolmogorov–Smirnov (KS) test is applied to identify any significant discrepancies in the distributions attributable to each batch effect. The feature set that shows the minimal distributional disparities is then chosen for further harmonization, and the associated batch effect is removed from the list for subsequent iterations. This process continues until all identified batch effects have been systematically addressed. The final harmonized feature set is rigorously analyzed to confirm that it exhibits no significant distribution differences when stratified by the original batch effects [19]. This meticulous approach to harmonization is vital to ensure the accuracy and reliability of our analysis, particularly in assessing the temporal changes in nodules across CT scans. We also performed the principal component analysis (PCA) to assess the impact of the Nested ComBat harmonization process on radiomic features.

### 2.7 Machine Learning Models

In this study, we employed nine ML models to evaluate the predictive power of six sets of original and harmonized radiomic features for lung cancer risk prediction. The models used were Random Forest (RF), Decision Tree (DT), Gaussian Naive Bayes (GNB), Support Vector Classifier (SVC), AdaBoost (Ada), XGBoost (XGB), Gradient Boosting (GB), Logistic Regression (LR), and K‐Nearest Neighbors (KNN). These models were chosen for their diverse approaches to handling classification tasks, allowing us to comprehensively assess the effectiveness of the feature sets in predicting lung cancer risk. These classification‐based models were implemented using the scikit‐learn package in Python, a library that easily facilitates the application of numerous ML algorithms. This streamlined the integration of various predictive modeling techniques into our analysis pipeline.

### 2.8 Feature Reduction and Selection Strategies

In high‐dimensional datasets, reducing and selecting features effectively is essential. This approach ensures that the models are both accurate and practical by avoiding features that do not contribute to or could introduce noise to our predictive models [42]. Our approach to handling this challenge involved a two‐step process aimed at refining the feature set for lung cancer risk prediction. Initially, we employed the Least Absolute Shrinkage and Selection Operator (LASSO) method to decrease the dimensionality of our dataset. LASSO is particularly suited for this task because it not only reduces the number of features but also helps in enhancing the model's interpretability by eliminating less important variables. Following the initial reduction, we applied five distinct feature selection techniques to identify the most relevant features. These methods included:

- *F*‐test: This method evaluates the individual features based on their statistical significance to the target variable using the analysis of variance (ANOVA) *F*‐value.
- Mutual Information (MI) [43]: This method assesses the mutual dependence between variables, helping to determine the amount of information shared between each feature and the target variable. This measure quantifies the amount of information that one variable reveals about another.
- ReliefF (RL), Surf (SF), and MultiSurf (MSF) [44, 45, 46]: ReliefF is effective in identifying relevant features and assigning a real‐valued score to each feature, indicating its relevance. Over time MSF and SF methods have been developed to enhance their applicability to various data types and analytical scenarios. These variants are incorporated in scikit‐rebate, a comprehensive Python package that supports a range of classification and regression tasks.

### 2.9 Balancing the Dataset

To address the challenge of our unbalanced 2:1 nested case–control dataset and to achieve a balanced dataset, we employed the Synthetic Minority Oversampling Technique (SMOTE) [47] implemented in Imbalanced‐learn, an open‐source MIT‐licensed library, relying on scikit‐learn package in Python. This oversampling method is specifically designed to enhance the representation of minority classes in datasets that are not balanced. Unlike simple random oversampling that duplicates existing samples, SMOTE generates synthetic samples by interpolating between several minority class instances. This technique not only helps in creating a more balanced dataset but also potentially improves the performance of ML models by providing a richer and more diverse set of training examples. In total, we utilized SMOTE in 135 models for each category of features: T0, delta, and T0 + delta. These models were generated through combinations of nine ML models and five feature selection methods, applied to three types of non‐harmonized, TC‐based, and KT‐based features. The results were compared to those obtained using the original unbalanced dataset.

### 2.10 Analysis Workflow

**FIGURE 2:**
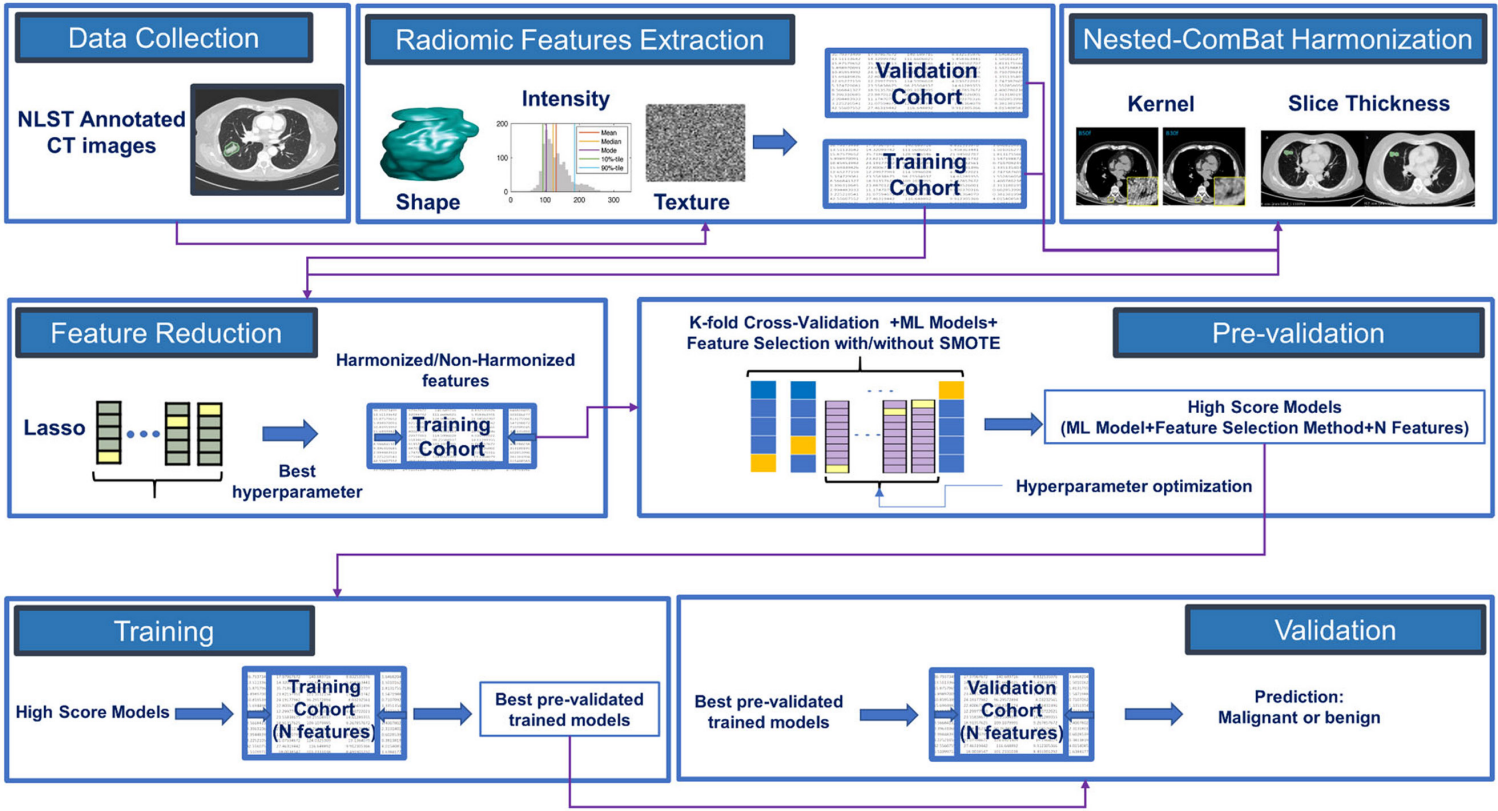
Workflow of the radiomic‐based predictive models in this study.

The pipeline of the radiomic‐based predictive models in our study presented in Figure 2 included three main steps: preprocessing, pre‐validation (training), and validation.

#### 2.10.1 Preprocessing

1. *Standardization of features:* All features in training and validation cohorts were normalized to have a mean of zero and a standard deviation of one.
2. *Feature reduction:* The number of features was effectively reduced by applying LASSO, which was optimized using a grid search using a 5‐fold cross‐validation with 10 times of repetition on the training dataset. The excluded features were those that received a zero coefficient from LASSO.

#### 2.10.2 Pre‐Validation Phase

In this step, we evaluated nine ML models using the training dataset applying a 5‐fold cross‐validation process that was repeated 10 times for robustness. During each fold, we varied the number of features from three up to the total identified by LASSO. These features were selected using five distinct feature selection methods, and the dataset was balanced using SMOTE in each scenario. We calculated the area under the curve (AUC) for each model configuration across all folds. The model configuration achieving the highest mean cross‐validation AUC score for each combination of ML algorithm and feature selection method was chosen for further analysis. To ensure the robustness of our models and their performance across various datasets, we performed hyperparameter tuning using GridSearchCV from the scikit‐learn library in Python. This approach allowed us to explore and select the optimal hyperparameters for each model configuration through cross‐validation.

#### 2.10.3 Validation Phase

The validation involved training the optimized models, each corresponding to a specific feature selection method and number of features, on the training cohort. Subsequently, their predictive performance was evaluated on the validation cohort using the AUC score as the evaluation metric.

## 3 Results

### 3.1 Study Population Characteristics

**FIGURE 3:**
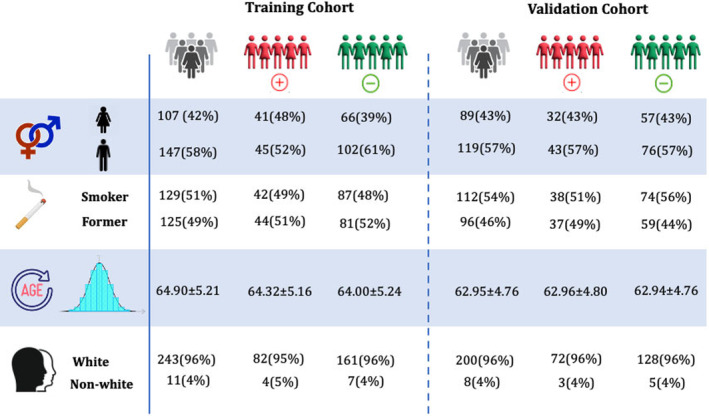
Clinical characteristics of training and validation cohorts used in the study.

Figure 3 outlines the demographic details of the training and validation cohorts used in this study, categorized by positive and negative cases.

The cohorts were established in such a way that the negative cases matched the positive cases in terms of age, sex, race, and smoking status, ensuring minimal differences in the distribution of these characteristics across the training and validation cohorts as well as between the positive and negative cases. Across the cohort of 462 samples, the individuals have an average age of 63.6 ± 5.0 years. Specifically, the training cohort consists of 254 individuals with an average age of 64.0 ± 5.2 years, while the validation cohort includes 208 individuals, averaging an age of 62.9 ± 4.8 years. Regarding smoking status, 51% of the patients in the training cohort and 54% in the validation cohort are smokers, compared to 49% and 46% who are non‐smokers, respectively. To validate the matching process, statistical tests were conducted to assess differences between positive and negative cases in age, sex, race, and smoking status. The *t*‐test for age revealed a *p*‐value of 0.72, indicating no significant difference between groups. The chi‐square test for sex produced a *p*‐value of 0.41, suggesting no significant association. For race, the chi‐square test yielded a *p*‐value of 0.06, indicating a trend toward significance. Finally, the chi‐square test for smoking status resulted in a *p*‐value of 0.50, showing no significant difference. These results confirm that the cohorts were successfully matched, with minimal differences in demographic and clinical characteristics.

### 3.2 Radiomic Feature Extraction

Using the Pyradiomics pipeline, out of 1231 radiomic features, 113 features categorized into shape‐based (27 features), intensity‐based (18 features), and texture‐based (68 features) were extracted directly from the original CT images, while 1118 intensity and texture‐based features were extracted from derived images that were obtained using various filters: 688 features using a Wavelet filter, and 86 features each from LoG, Gradient, Square, Square‐root, and Exponential filters. Radiomic features were extracted from 1694 CT images available at T0, T1, and T2. Not all images were included in the training and validation cohorts. For instance, some images were produced using two different reconstruction kernels for the same patient. Furthermore, features extracted from T2 were also not utilized in ML analyses. Nevertheless, to enhance the robustness of our data for effective harmonization, we extracted radiomic features from as many CT scans as possible. This approach aimed to enrich our dataset with a sufficient number of samples from each TC and KT, enhancing the accuracy and effectiveness of the harmonization process for more precise feature adjustment.

#### 3.2.1 Harmonization of Radiomic Features

**FIGURE 4:**
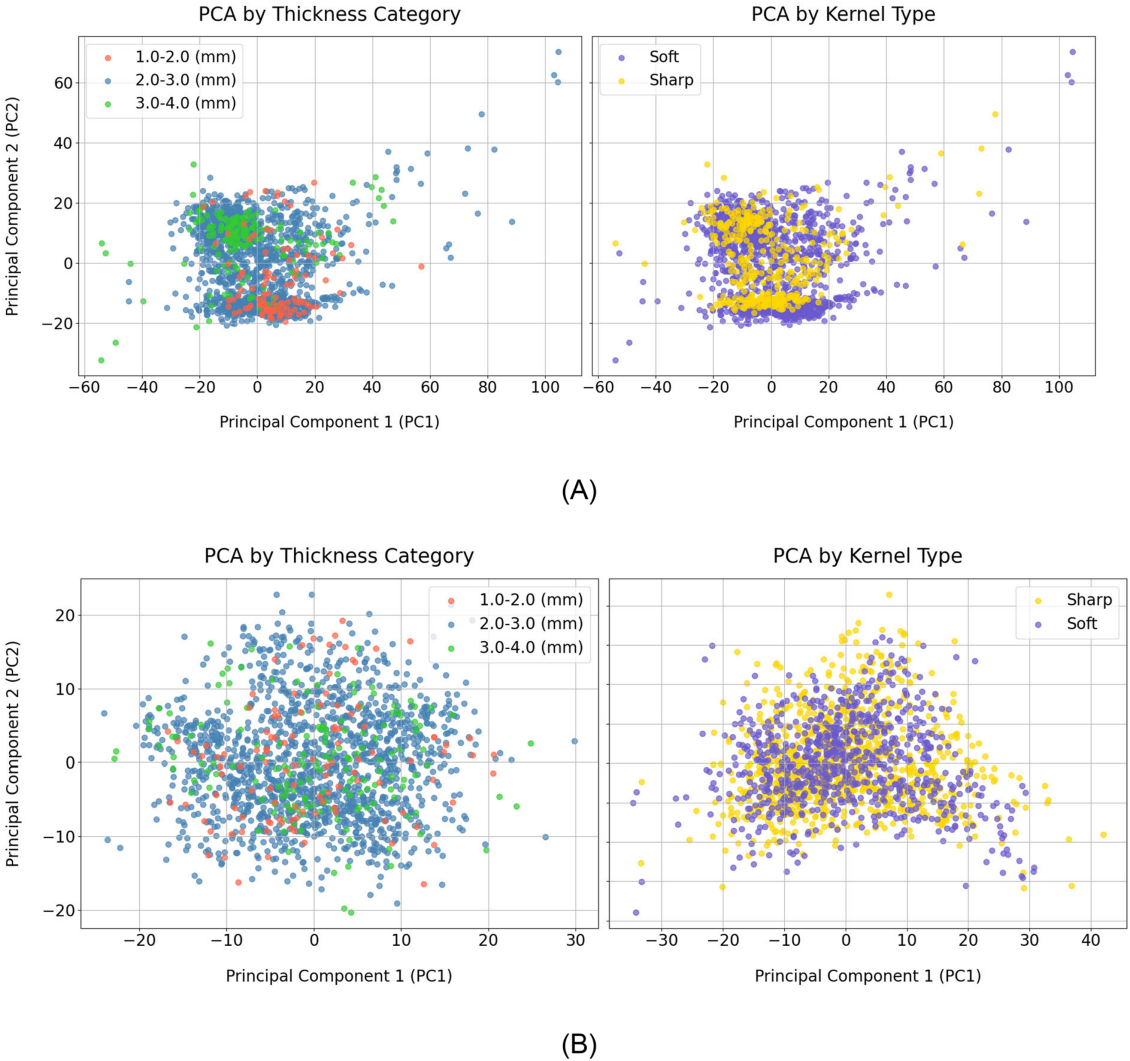
The representation of TC (left) and KT (right) imaging parameters using principal component analysis (PCA) of radiomic features before (A) and after (B) Nested Combat harmonization.

Radiomic features were extracted from 1689 out of the available 1694 CT images available, with five images excluded due to missing clinical data, slice thickness, or reconstruction kernel information. The slice thicknesses across these images ranged from 1.0 to 3.2 mm, with a median of 2.5 mm. For harmonization purposes, the images were stratified into three categories based on slice thickness: 1.0–2.0 mm, 2.0–3.0 mm, and 3.0 to 4.0 mm, resulting in distributions of 112, 1410, and 164 samples, respectively. Additionally, images were classified by reconstruction kernels into sharp (389 images) and soft (1300 images). After the harmonization of the radiomic features based on the TC and KT independently, and to better understand the impact of Nested ComBat harmonization on these features, we utilized principal component analysis (PCA). This approach facilitates the projection and visualization of high‐dimensional data. Figure 4A,B illustrates the distribution of radiomic features based on PCA results, before and after harmonization. The left panel of Figure 4A highlights the groups by TC while the right panel presents the distinct groups based on KT before harmonization. As can be seen in both panels of Figure 4A, before harmonization clustering by TC or KT is observable, though the clusters are not significantly distinct. The left panel in Figure 4B presents the distribution of the features after harmonization based on TC while the right panel presents it when harmonization is done based on KT. Both panels highlight how the harmonization process which was done on TC and KT independently, effectively reduces discrepancies caused by different TC or KT groups, leading to a more homogeneous dataset with reduced variabilities between the TC or KT clusters.

### 3.3 Performance of Models

In total, we developed 270 models for each category of extracted radiomic features‐ T0, delta, and T0 + delta‐ by integrating nine ML models and five feature selection methods which were carried out on non‐harmonized, TC‐ and KT‐based harmonized features with and without SMOTE. The maximum number of features used in developing ML models, determined through the LASSO feature reduction technique, ranged from 14 to 46. This variability was dependent on the feature category and the type of harmonization applied. The importance of features selected by LASSO along with LASSO curves for each of these categories has been plotted in Supplementary file, Figures S1–S9. The AUC ranges varied based on the use of SMOTE, harmonization, and type of harmonization. The median performance of each of the nine ML models over all five feature selection methods using T0, delta, and T0 + delta feature sets has been presented in Figure S10A–C in the supplementary file.

For the T0 features, the range of AUC on the validation dataset without SMOTE was [0.56, 0.72] for non‐harmonized features, [0.54, 0.69] for KT‐based harmonized features, and [0.51, 0.69] for TC‐based harmonized features. With the application of SMOTE, the corresponding ranges were [0.60, 0.68], [0.54, 0.69], and [0.59, 0.69]. The highest AUC observed was 0.72 on the validation dataset, paired with a training score of 0.73 ± 0.07, achieved by the GB model using non‐harmonized features and the Surf feature selection method without SMOTE. On the other hand, the lowest AUC on the validation dataset was 0.51, with a training dataset score of 0.72 ± 0.04, obtained using an SVC with the *F*‐test feature selection method on TC‐based harmonized features without SMOTE. The application of SMOTE improved the minimum AUC when each of the non‐harmonized, KT‐based, and TC‐based harmonized features were employed; however, the maximum AUC did not improve by SMOTE.

Regarding the delta features, AUCs on the validation set without SMOTE ranged from [0.60, 0.73] for non‐harmonized features, [0.64, 0.72] for KT‐based, and [0.60, 0.74] for TC‐based harmonized features. When SMOTE was implemented, these AUCs altered to [0.61, 0.76], [0.62, 0.74], and [0.64, 0.76] respectively. The maximum AUC achieved was 0.76 on the validation set, corresponding to a training score of 0.85 ± 0.03, found in the GB model with TC‐based harmonized features and the MultiSurf selection method with SMOTE. The combination of the RF model with the MI feature selection method and SVC with the Surf feature selection method also resulted in the maximum AUC of 0.76 on the validation dataset using non‐harmonized features with SMOTE. The minimum AUC of 0.60 on the validation dataset was noted in the DT model employing the *F*‐test feature selection method with non‐harmonized features without SMOTE, achieving a training performance of 0.81 ± 0.03. Similarly, a DT model utilizing the MultiSurf feature selection method with TC‐based harmonized features, also without SMOTE, recorded the same AUC on validation, with a training performance of 0.84 ± 0.06.

For the combined T0 + delta features, the analysis of AUCs on the validation dataset without the use of SMOTE showed a range of [0.64, 0.73] for non‐harmonized features, [0.63, 0.74] for KT‐based harmonized features, and [0.62, 0.73] for TC‐based harmonized features. Upon applying SMOTE, these AUCs shifted to ranges of [0.58, 0.74], [0.60, 0.76], and [0.56, 0.76]. The highest AUC achieved on the validation dataset was 0.76, obtained in two scenarios: with an SVC model using KT‐based harmonized features and the *F*‐test feature selection method with SMOTE, and with an XGB model applying the MI feature selection method on TC‐based harmonized features with SMOTE, each yielding a training AUC of 0.86 ± 0.02. In contrast, the minimum AUC on the validation dataset was observed as 0.56, associated with a training score of 0.83 ± 0.05, derived from a DT model that utilized TC‐harmonized features and the *F*‐test feature selection method with SMOTE. This indicates variability in model performance based on feature selection and harmonization approaches, underlining the importance of choosing the right combination for optimal prediction.

**FIGURE 5:**
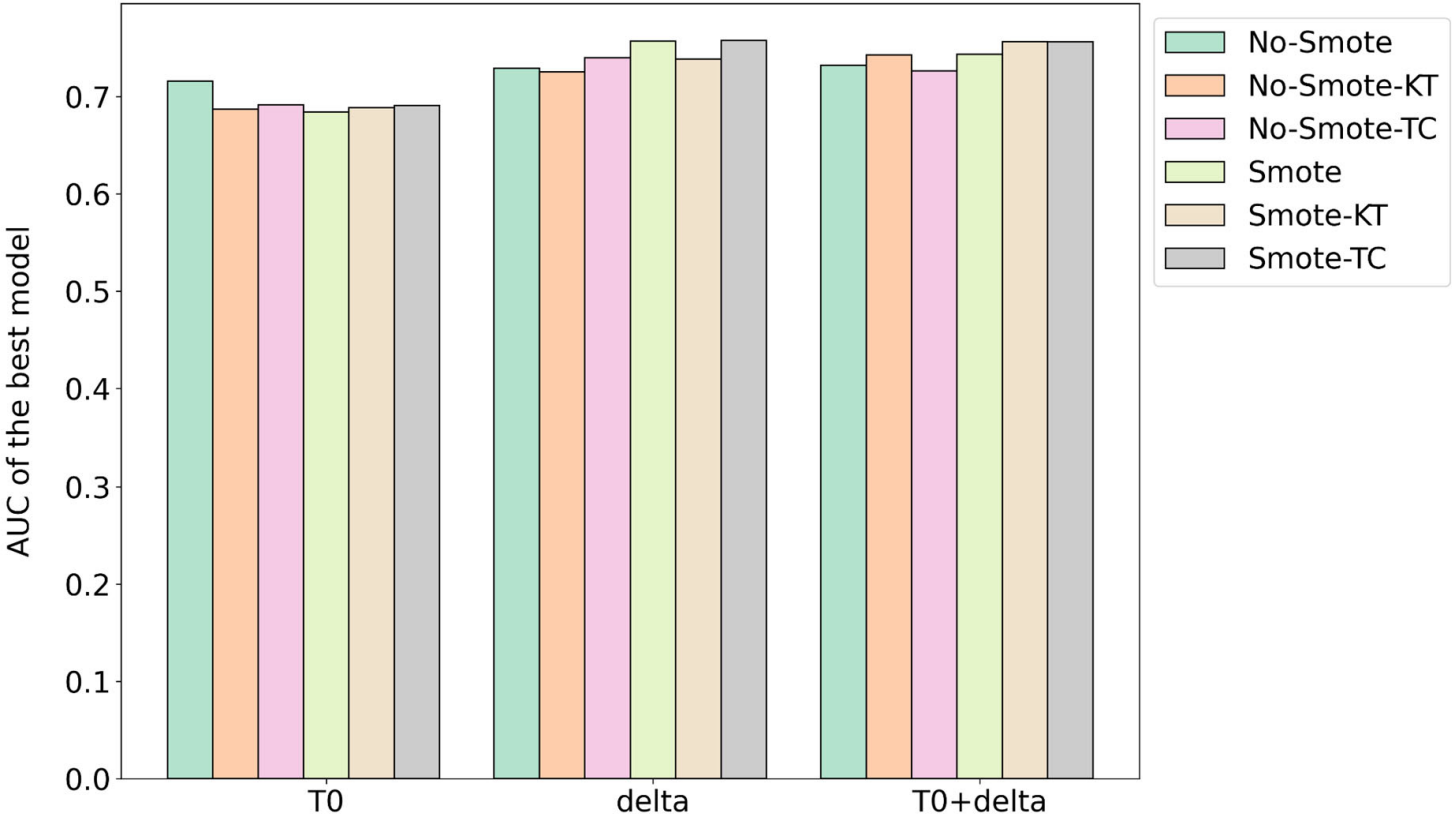
Comparison of the AUC for the best model using different harmonized and non‐harmonized feature sets with and without SMOTE.

Figure 5 presents a comparison of the best‐performing models on the validation cohort for each feature set (T0, delta, and T0 + delta), considering both scenarios with and without Nested ComBat harmonization based on KT and TC. The results highlight that utilizing SMOTE generally enhances outcomes; specifically, harmonization based on TC improves results across all feature sets when SMOTE is applied, while harmonization based on KT does not enhance performance for the delta features. Notably, the highest‐performing model, which uses SMOTE with delta features and TC‐based harmonization, achieved an AUC score of 0.76. To further support these observations, ANOVA was conducted to test for significant differences between AUCs of models utilizing SMOTE and not utilizing SMOTE across the different feature sets. The results indicate that there is no significant difference between them for the T0‐based models (*p* = 0.335). For the delta‐based models, a *p*‐value of 0.062 suggests a close‐to‐significant difference. Moreover, in the case of the models using T0 + delta features, a significant difference was found (*p* = 0.046), indicating that the combination of T0 and delta features significantly affects model performance when using SMOTE.

**FIGURE 6:**
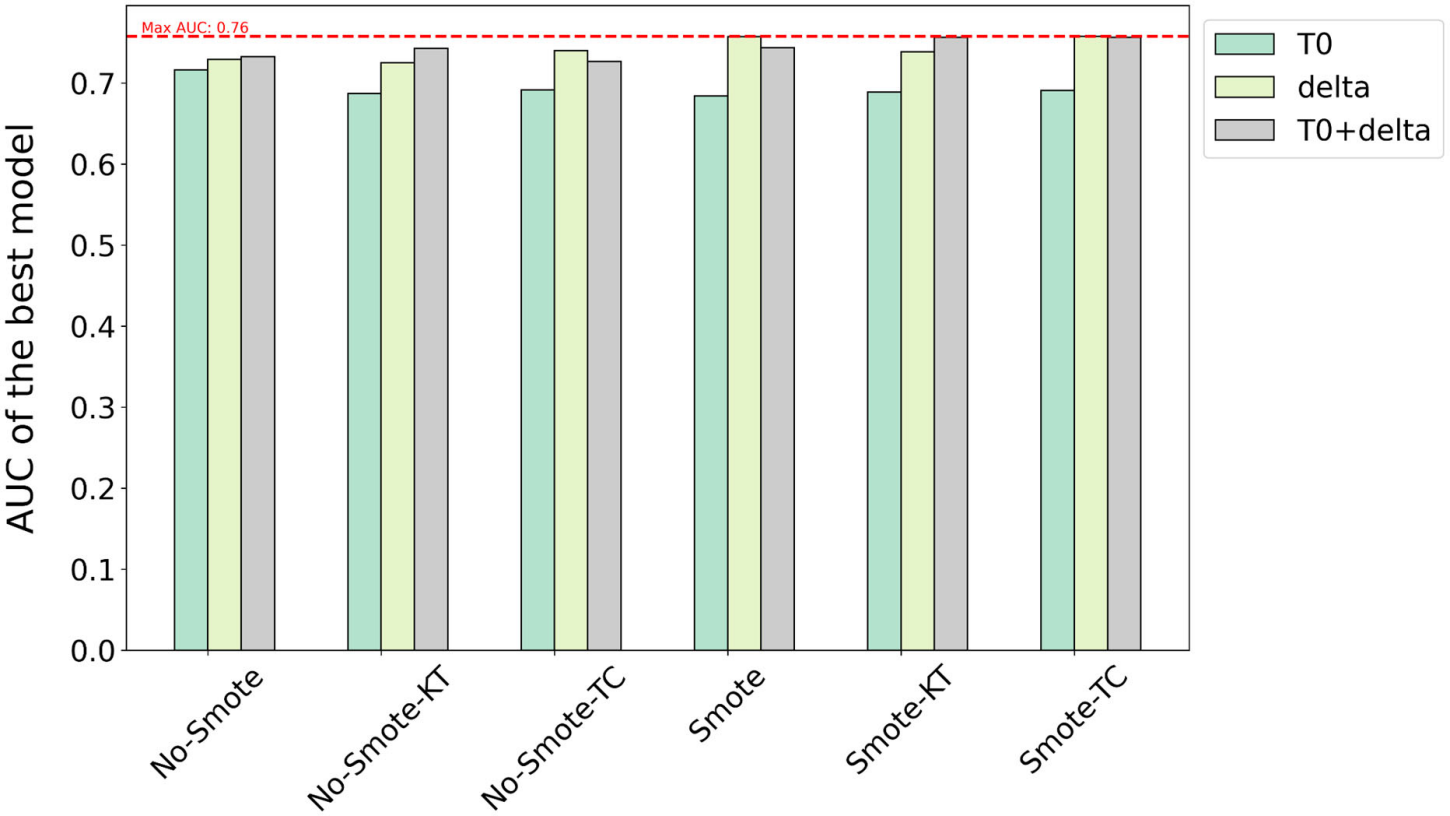
Comparison of the AUC for the best model within each pipeline using different feature sets.

Additionally, Figure 6 extends this comparison across different feature sets. The results demonstrate that models using delta features consistently outperform those using only T0 features, regardless of the SMOTE application. This is further supported by a statistically significant difference between the AUC scores for models using T0 and delta features, with an ANOVA test yielding a *p*‐value of less than 0.0001, confirming that the difference is highly significant. However, the addition of delta features to T0 does not yield improvements over using delta features alone, as indicated by an ANOVA test comparing delta and T0 + delta models, which produced a non‐significant *p*‐value of 0.823. Similarly, a significant difference is observed between models using only T0 features and those using T0 + delta features (*p* < 0.0001), suggesting that while adding delta features enhances performance compared to T0 alone, it does not outperform delta features used independently.

## 4 Discussion

While lung cancer screening has its benefits, LDCT imaging often detects intermediate nodules, most of which are benign. This creates a significant challenge due to the lack of effective clinical decision‐making tools for accurately predicting lung cancer risk. This study was aimed to develop and validate ML models that leverage radiomic features extracted from CT images to predict lung cancer risk. Our unique approach involved a nested case–control analysis of the NLST, incorporating both baseline and delta radiomic features, with and without harmonization. Additionally, we used SMOTE to address the class imbalance. In CT scans, slice thickness can range from sub‐millimeter to over 5 mm, depending on the area being imaged. Different reconstruction kernels, such as “hard” or “sharp” for higher spatial resolution and “soft” for reduced noise, present a trade‐off between spatial resolution and noise levels. To harmonize the data, we applied the ComBat harmonization method, which adjusts for variations in imaging parameters like slice thickness and reconstruction kernel. Originally introduced in 2007 [29] to address batch effects in genomics, ComBat can be directly applied to calculated features, bypassing the necessity to reanalayze the images. This eliminates the need to modify preprocessing parameters, which might have been required for pre‐harmonization methods utilized prior to feature extraction. Studies have shown that it is highly effective at reducing and removing batch effects while increasing precision and accuracy compared to other popular batch effect removal methods [25, 48].

After harmonizing the radiomic features, PCA visualization confirmed that the harmonization effectively resulted in a more uniform distribution of radiomic features, minimizing clustering based on slice thickness and reconstruction kernel. This uniformity is crucial for ensuring the generalizability of ML models across different imaging parameters. The performance of post‐harmonization models was influenced by the distribution and distinction between different classes of slice thickness and reconstruction kernel. While PCA revealed clustering for slice thickness categories before harmonization, it did not show significant clustering for reconstruction kernels. Since harmonization eliminated these clusters, making the models more generalizable across different imaging parameters, it was expected that model performance would not improve as much with KT‐based harmonized features, where clusters were less prominent, compared to TC‐based harmonized features. This likely explains the equal or superior performance of TC‐based harmonized features over KT‐harmonized features when SMOTE was applied. For T0 features, the best‐performing model achieved an AUC of 0.69 using either TC‐ or KT‐based harmonized features. For delta features, the AUC reached 0.76 for TC‐based harmonized features, compared to 0.74 for KT‐based harmonized features. When using T0 + delta features, an AUC of 0.76 was achieved with both TC‐ and KT‐based harmonized features.

In examining the effectiveness of harmonizing radiomic features, our results indicated that the models did not show a significant improvement post‐harmonization, regardless of whether SMOTE was applied. This conclusion contrasts with some studies where harmonization techniques have yielded positive outcomes [25]. However, it is important to note that our findings are aligned with other research where ComBat harmonization did not significantly enhance the performance of radiomic models in predicting overall survival for patients with clear cell renal cell carcinoma [27]. These findings underscore the need for further investigation into the application of harmonization techniques for predictive models, highlighting the necessity to advance this field. These contrasting results highlight the complex nature of harmonization techniques and their varying effectiveness across different contexts and patient populations. Our findings emphasize the necessity for further investigation into the application of harmonization methods in predictive modeling, particularly regarding the influence of imaging parameters. By advancing our understanding in this area, we can better assess the potential benefits of harmonization for improving the accuracy and generalizability of predictive models.

Models that utilized delta radiomic features exhibited better performance compared to those using only baseline features, aligning with existing literature [8]. This confirms that temporal changes in radiomic features are valuable for capturing the progression of potential lung cancer, regardless of whether the dataset is balanced or imbalanced. The application of SMOTE significantly improved the AUC performance metric across all models when T0 + delta features were employed. Notably, with SMOTE, the TC‐based harmonization always enhanced model performance, whereas KT‐based features did not improve models when delta features were utilized. This suggests that the combination of SMOTE and harmonization based on slice thickness in CT images could be effective for predictive modeling. These results are consistent with previous studies. The effectiveness of applying SMOTE on the performance of radiomics‐based predictive models has been explored in several studies [8, 49]. It has been demonstrated that the AUCs of radiomics‐based models using SMOTE are significantly improved compared to those using the original dataset. Additionally, SMOTE has been shown to outperform other subsampling methods [49].

Reflecting the discussion by Cherezov et al. [8], who used the same NLST subsamples, both the limitation and strength of this study are inherent in the data itself. One limitation is the use of a relatively small subsample, despite the availability of more extensive data that could potentially enhance model performance. Utilizing radiomic features for predictive models requires nodule segmentation by radiologists, a task that remains time‐consuming. In this work, segmentation data were provided by a previous study [32]. In the future, leveraging auto segmentation tools could allow us to extract radiomic features from a larger number of CT scans using NLST data, facilitating further investigations into radiomic‐based biomarkers for lung cancer risk prediction. A notable strength of this study was the careful matching of demographics such as age, smoking status, race, and gender between positive and negative cases, and between training and test cohorts. Additionally, these covariates were considered during the harmonization process, ensuring a more accurate and unbiased evaluation of the predictive models using harmonized features. These approaches helped reduce the potential impact of confounding variables, enhancing the effectiveness of the subsample used. However, there may still be hidden confounders that could affect the ability of radiomic features to differentiate between positive and negative cases. In future work, incorporating demographic risk factors into the pipeline by adding them to the radiomic features might help retain valuable data, further improving the development of predictive models.

In conclusion, our study demonstrated that both delta radiomics and the combination of T0 + delta radiomics significantly improve the model's performance compared to using T0 radiomics alone. However, when applying SMOTE, only the combination of T0 + delta radiomics leads to a statistically significant improvement in performance, while delta radiomics alone does not show a similar significant effect. This indicates that the inclusion of T0 radiomics alongside delta radiomics enhances the impact of SMOTE on model performance, suggesting that the combined feature set benefits more from SMOTE application than individual feature sets.

While several diagnostic models for pulmonary nodules demonstrate superior performance, their effectiveness may be limited to specific demographic groups or clinical scenarios, potentially reducing their applicability to broader patient populations. For example, the Mayo Clinic model [50], which integrates three clinical and three radiographic features, has been widely employed since its introduction in 1997 and exhibits reasonable diagnostic accuracy, with an AUC of 0.832. However, concerns exist regarding its ability to accurately predict malignancy, particularly among low‐risk patients [51]. In a recent study by Chen et al. [51], the developed model achieved AUC values of 0.884 and 0.820 in the training and validation cohorts, respectively, outperforming existing methods, including the Mayo Clinic model. However, this study was limited by a relatively small sample size for benign nodules, comprising 72 benign pulmonary nodules versus 188 early‐stage malignant nodules, and it did not utilize balancing methods, which may hinder the generalizability of the findings. Furthermore, the cohort predominantly consisted of participants from a single ethnic group, highlighting the need for further validation in more diverse populations to ensure broader applicability of the results. In contrast, while the performance of our model may be lower, it has been designed to enhance generalizability across multiple aspects. Firstly, we employed harmonization techniques that ensure radiomic features remain consistent and robust across various scanners and institutions, thus enhancing the applicability of our models in multi‐institutional settings. Models trained on unharmonized features may exhibit overfitting to specific acquisition settings, limiting their generalizability to datasets with different imaging protocols. Our comparison of pre‐ and post‐harmonization features demonstrated that post‐harmonization models exhibited a more uniform distribution of radiomic features across varying scanner parameters, as confirmed by PCA visualization. The elimination of clusters related to these imaging parameters mitigates bias from acquisition variations, leading to more consistent performance when applied to data from diverse scanners. Additionally, our study emphasizes the interpretability of radiomic features. The inclusion of delta features, which capture the temporal change in lung nodules, enhances the model's ability to monitor lung cancer risk longitudinally, supporting effective patient management. By integrating demographic and clinical risk factors into the harmonization process, we minimized potential confounding effects from variables such as age, smoking status, race, and gender. This approach allows for a more accurate and unbiased evaluation of the predictive models, making the radiomic features more interpretable within real‐world clinical contexts. Furthermore, we strengthened our model by applying SMOTE to balance the dataset, which helps mitigate the impact of class imbalance on model performance. By augmenting the representation of benign nodules, we aim to improve the model's predictive capabilities and generalizability. In conclusion, the harmonization of radiomic features not only enhances the generalizability of our models but also improves their clinical interpretability, facilitating more reliable predictions of lung cancer risk.

Despite these insights, we recognize that our study was limited by the small subsample of NLST data, which raises questions about the generalizability of our findings to other populations. To address this, future research will involve applying our models to larger, multi‐institutional datasets and incorporating various modified ComBat harmonization techniques. These efforts will help validate our models and explore their applicability in broader, diverse settings. Additionally, while harmonizing radiomic features based on imaging parameters makes them generalizable across different institutions and scanners, further investigation is warranted. Future work should integrate various modified ComBat harmonization techniques such as the Gaussian Mixture Model (GMM)‐based method (GMM ComBat) where scans are split into groupings based on the shape of the distribution used for harmonization as a batch effect and subsequent harmonization by a known imaging parameter [19], M‐ComBat which allows transforming all features distributions to a chosen reference, instead of the overall mean, providing more flexibility, B‐ComBat which adds bootstrap and Monte Carlo for improved robustness in the estimation, and BM‐ComBat which combines both modifications [25]. Also, additional imaging parameters, such as different scanner types and models, will be considered to identify robust and reproducible predictive radiomic features from multi‐institutional CT scans. Such efforts will be instrumental in discovering reliable radiomic‐based biomarkers that can complement existing risk assessment standards and contribute to the early‐stage prediction of lung cancers.

## Author Contributions

**Leyla Ebrahimpour:** conceptualization (equal), data curation (lead), formal analysis (equal), methodology (equal), software (lead), writing – original draft (equal), writing – review and editing (equal). **Philippe Després:** conceptualization (equal), formal analysis (supporting), funding acquisition (equal), supervision (equal), writing – original draft (equal), writing – review and editing (equal). **Venkata S. K. Manem:** conceptualization (equal), funding acquisition (equal), methodology (equal), supervision (equal), writing – original draft (equal), writing – review and editing (equal).

## Conflicts of Interest

The authors declare no conflicts of interest.

## Supporting information


Data S1.

## Data Availability

We acknowledge the National Cancer Institute (NCI) for granting access to the radiological and clinical data from the LDCT arm via The Cancer Imaging Archive (TCIA).
