# Supplementary material for: Differential Radiomics‐Based Signature Predicts Lung Cancer Risk Accounting for Imaging Parameters in NLST Cohort
Source: Cancer Med. 2024 Oct 28;13(20):e70359. doi: 10.1002/cam4.70359 (PMC11513548; doi:10.1002/cam4.70359)
Supplement: Supplementary file 1 — Data S1. [file CAM4-13-e70359-s001.docx]

**Supplementary file**


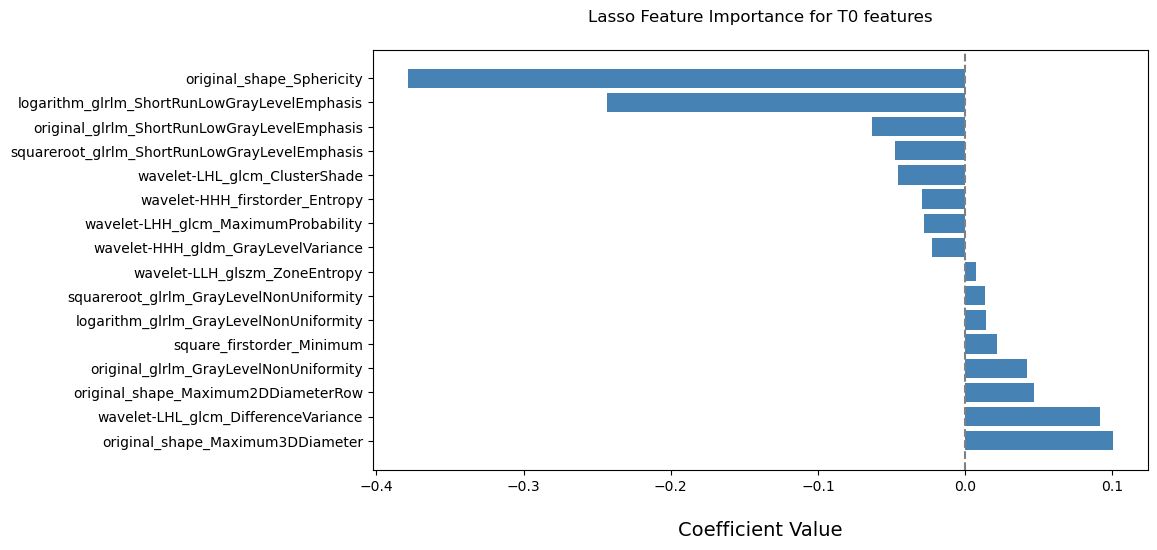


**(A)**


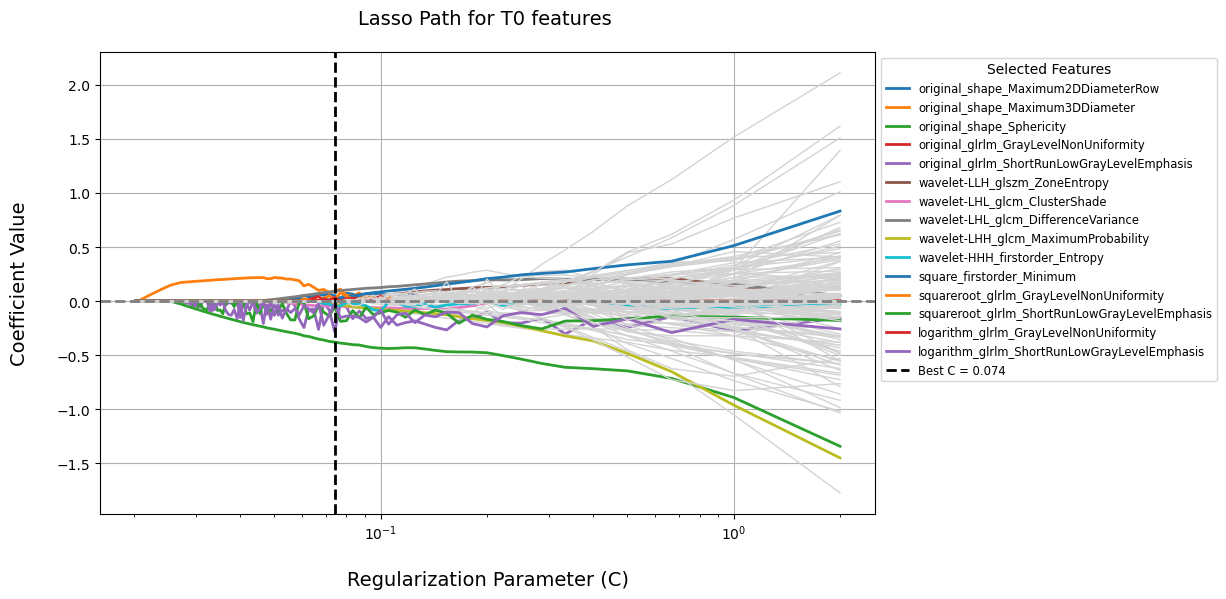


**(B)**

**Figure S1**- Feature importance (A) and Lasso curve (B) for 16 T0 not-harmonized radiomic features selected by LASSO.


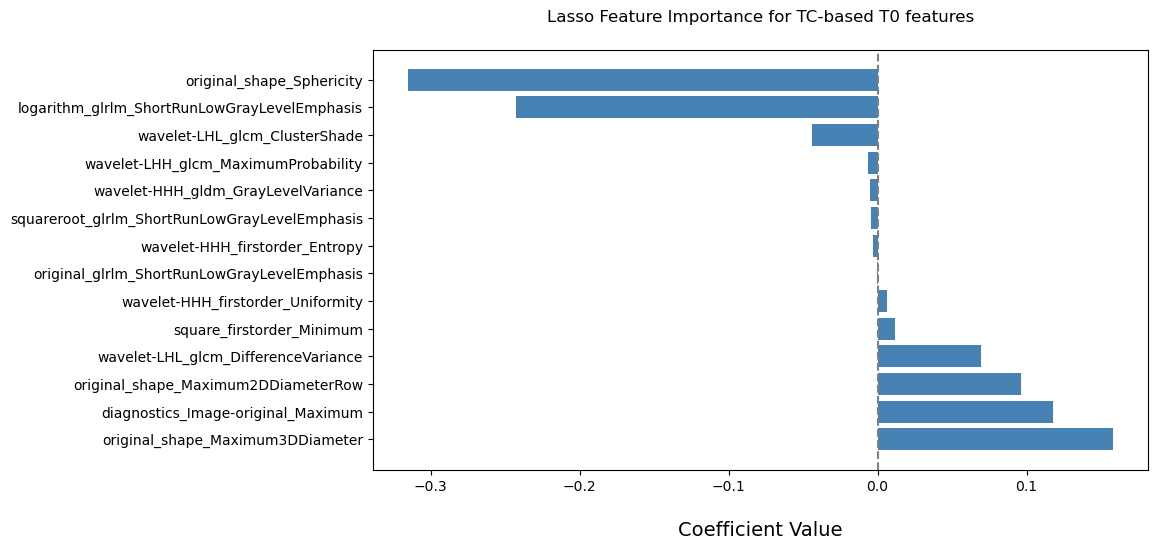


**(A)**


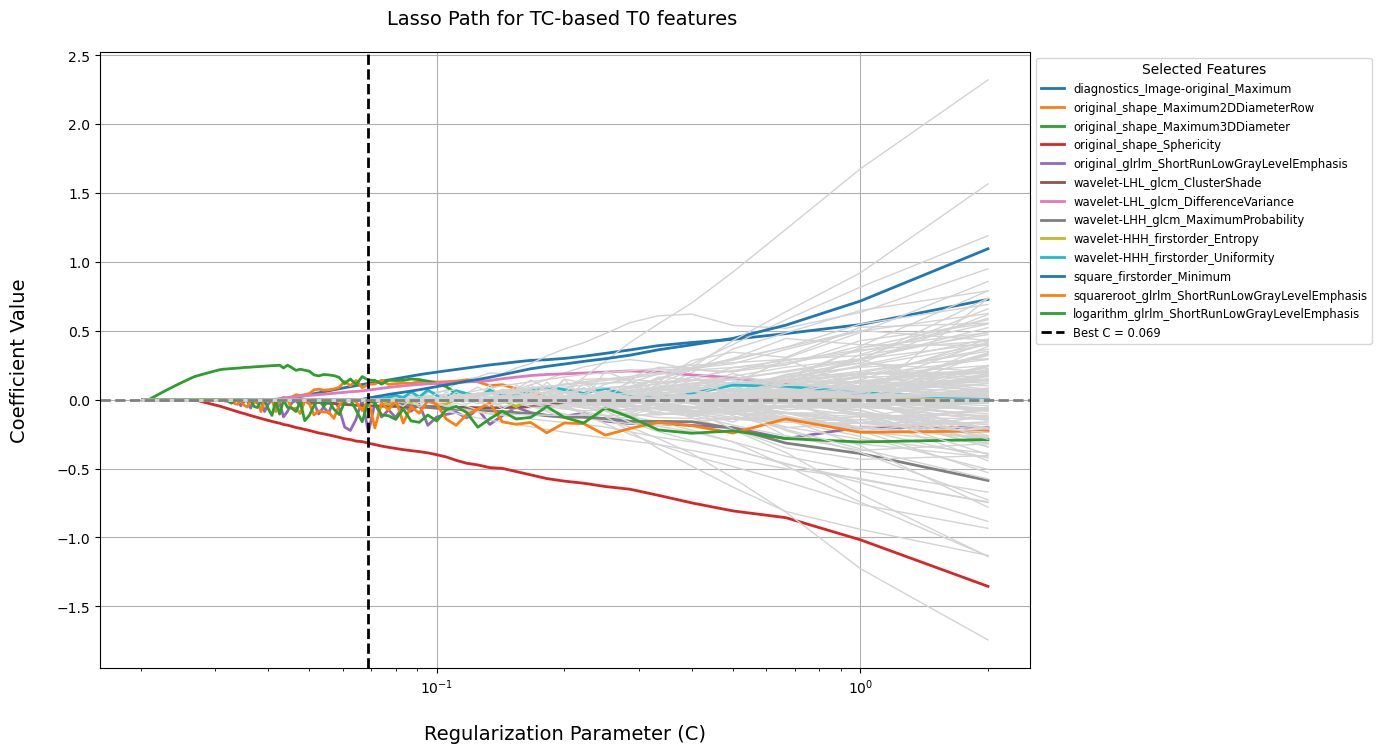


**(B)**

**Figure S2**- Feature importance (A) and Lasso curve (B) for 14 T0 TC-based radiomic features selected by LASSO.


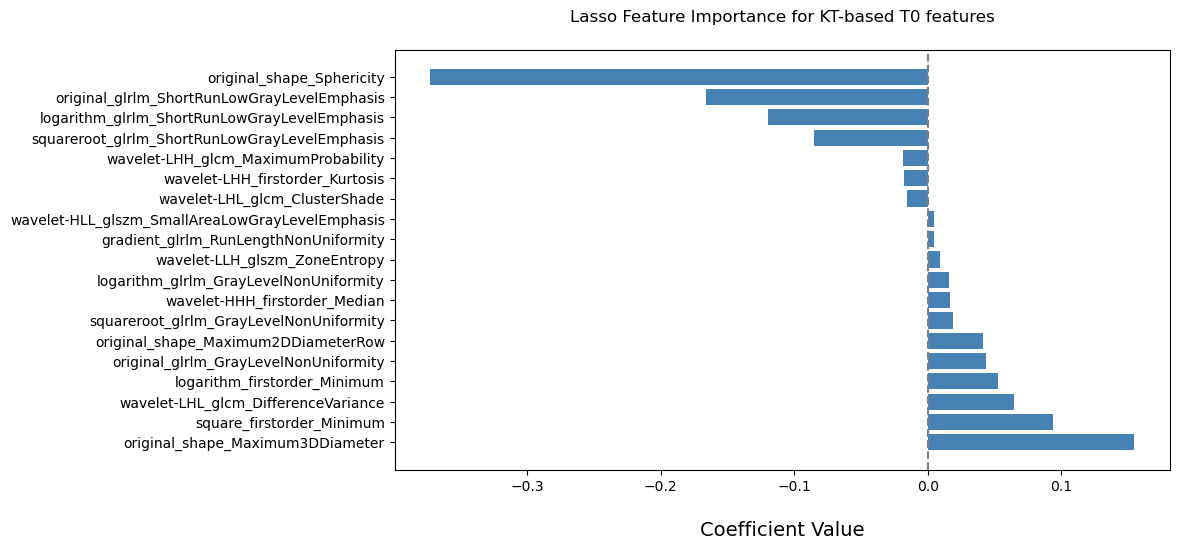


**(A)**


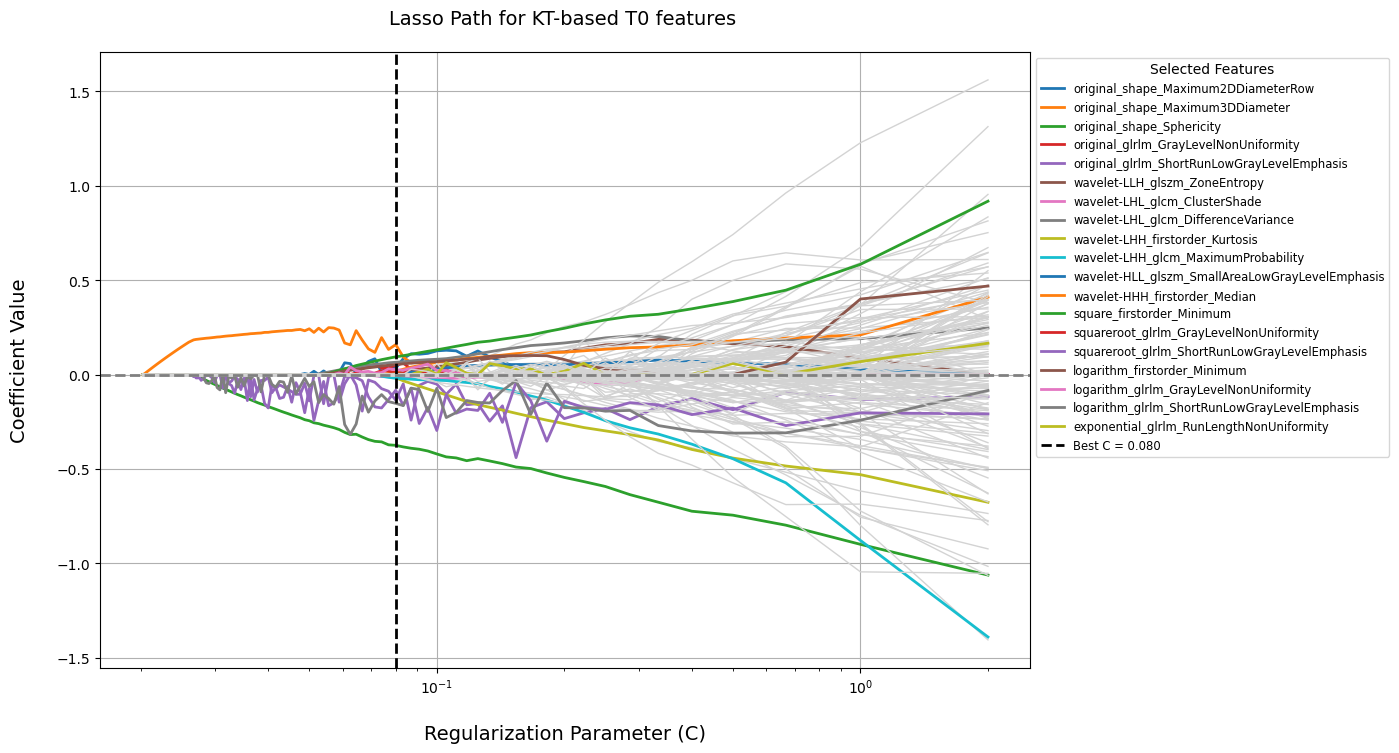


**(B)**

**Figure S3-** Feature importance (A) and Lasso curve (B) for 19 T0 KT-based radiomic features selected by LASSO.


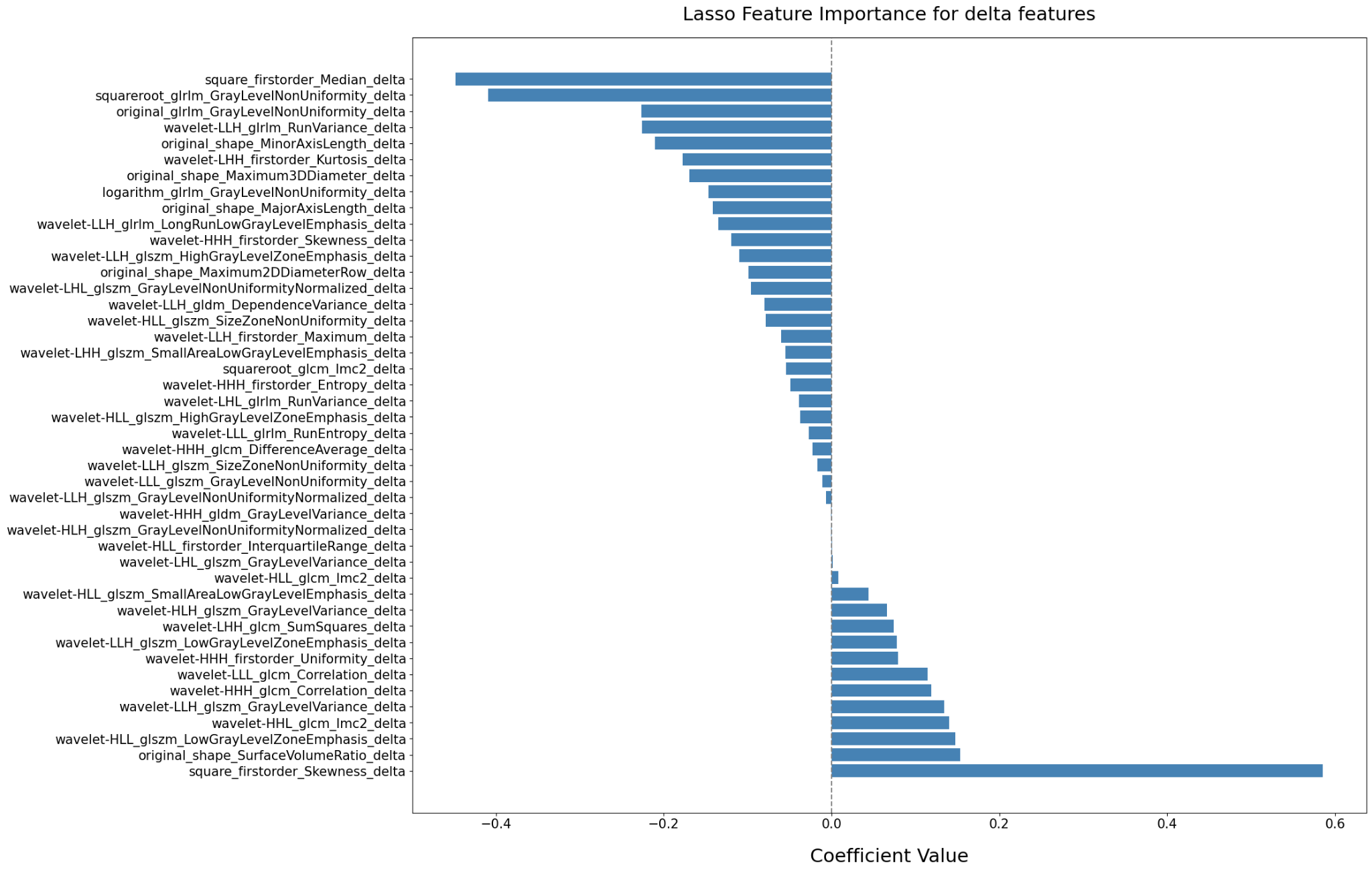


**(A)**


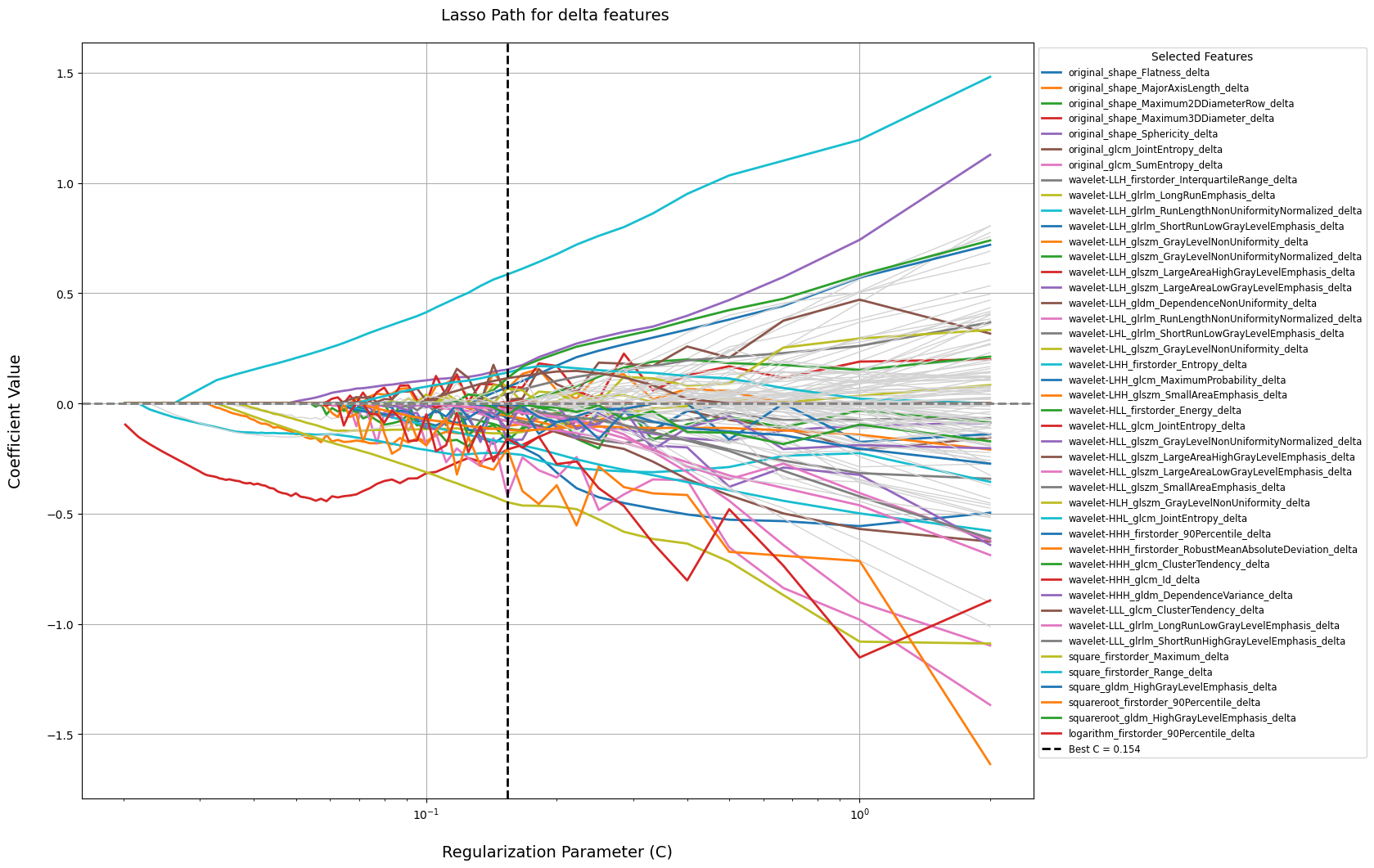


**(B)**

**Figure S4-** Feature importance (A) and Lasso curve (B) for 44 delta not-harmonized radiomic features selected by LASSO.


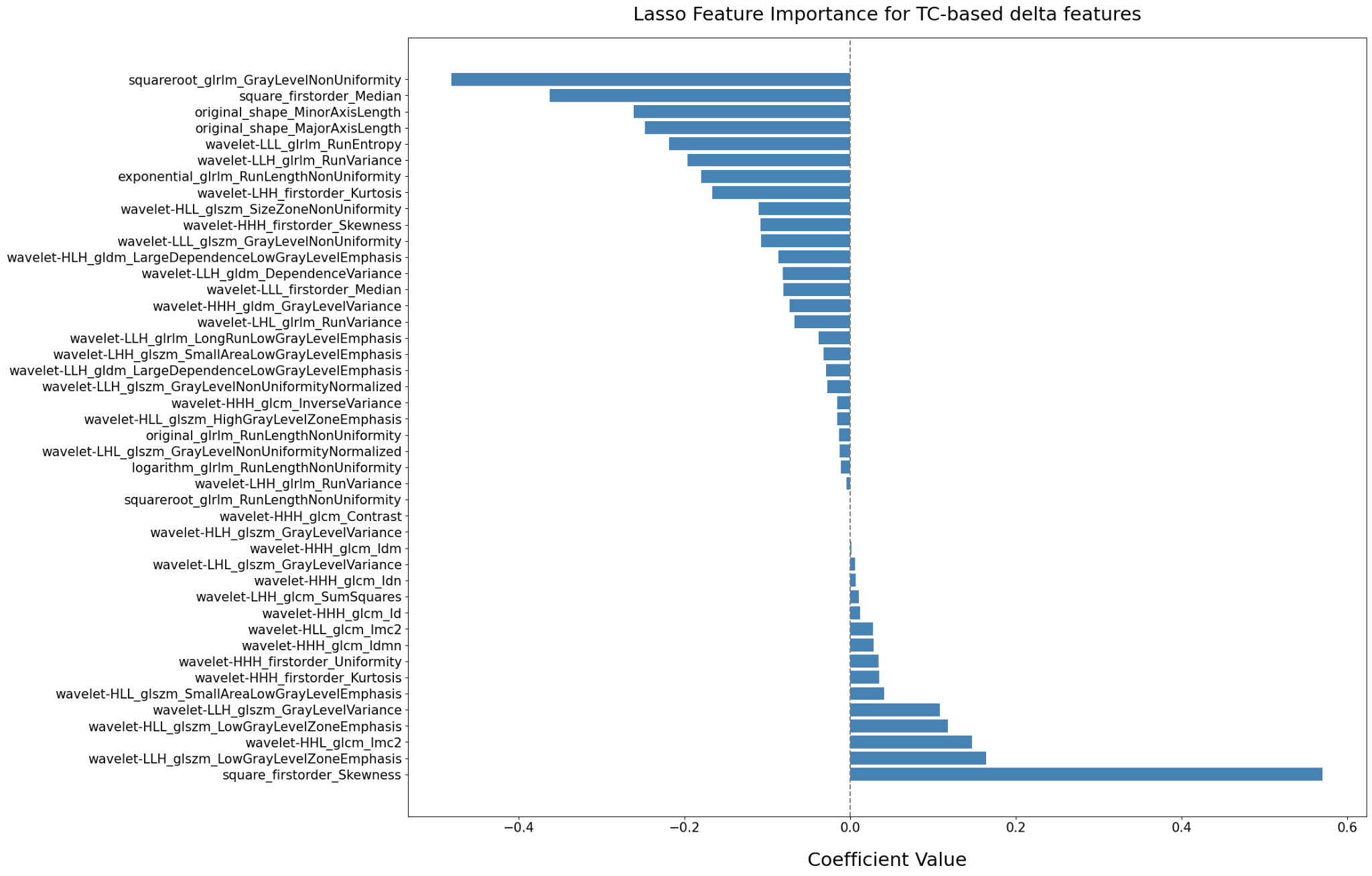


**(A)**


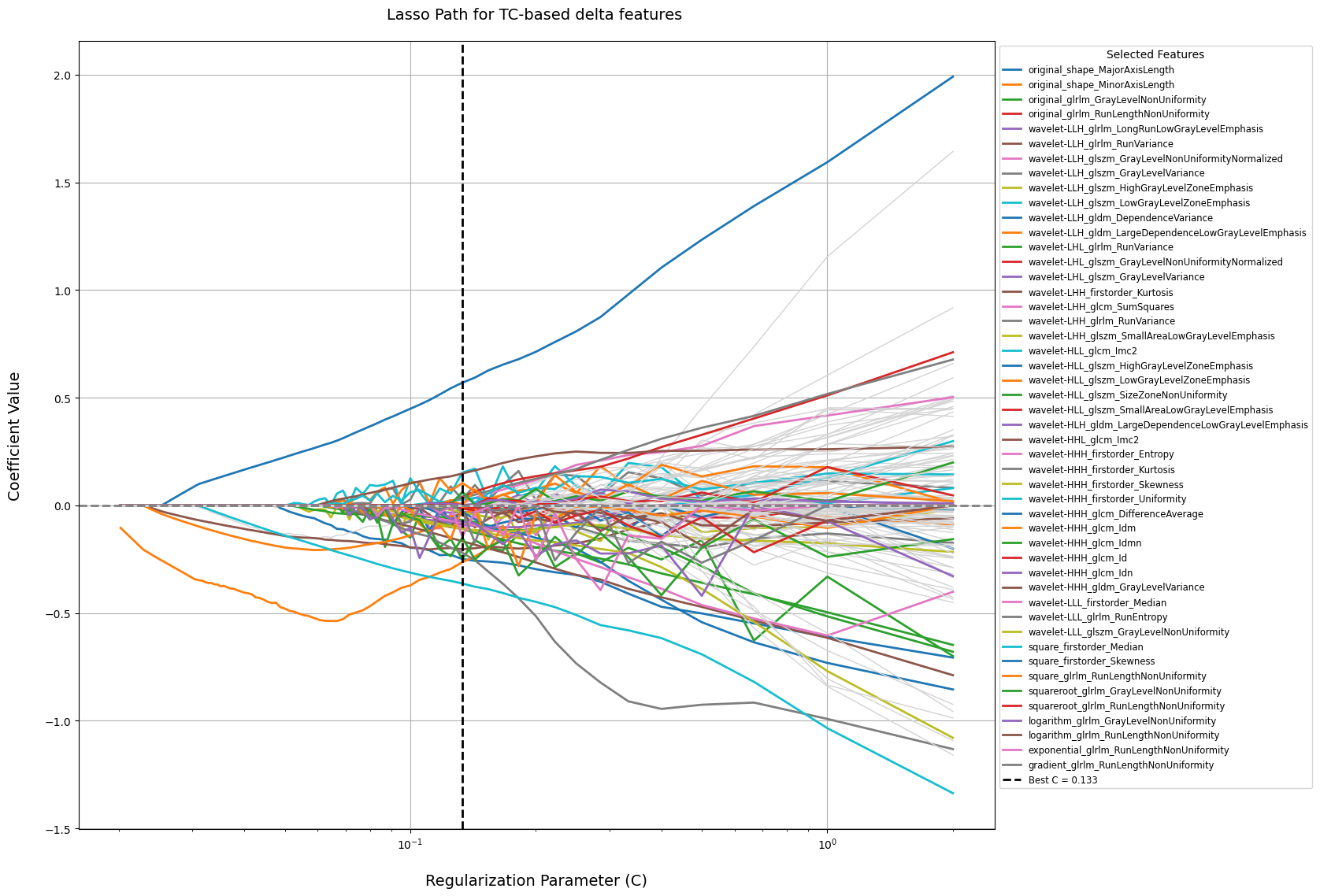


**(B)**

**Figure S5-** Feature importance (A) and Lasso curve (B) for 22 delta TC-based radiomic features selected by LASSO.


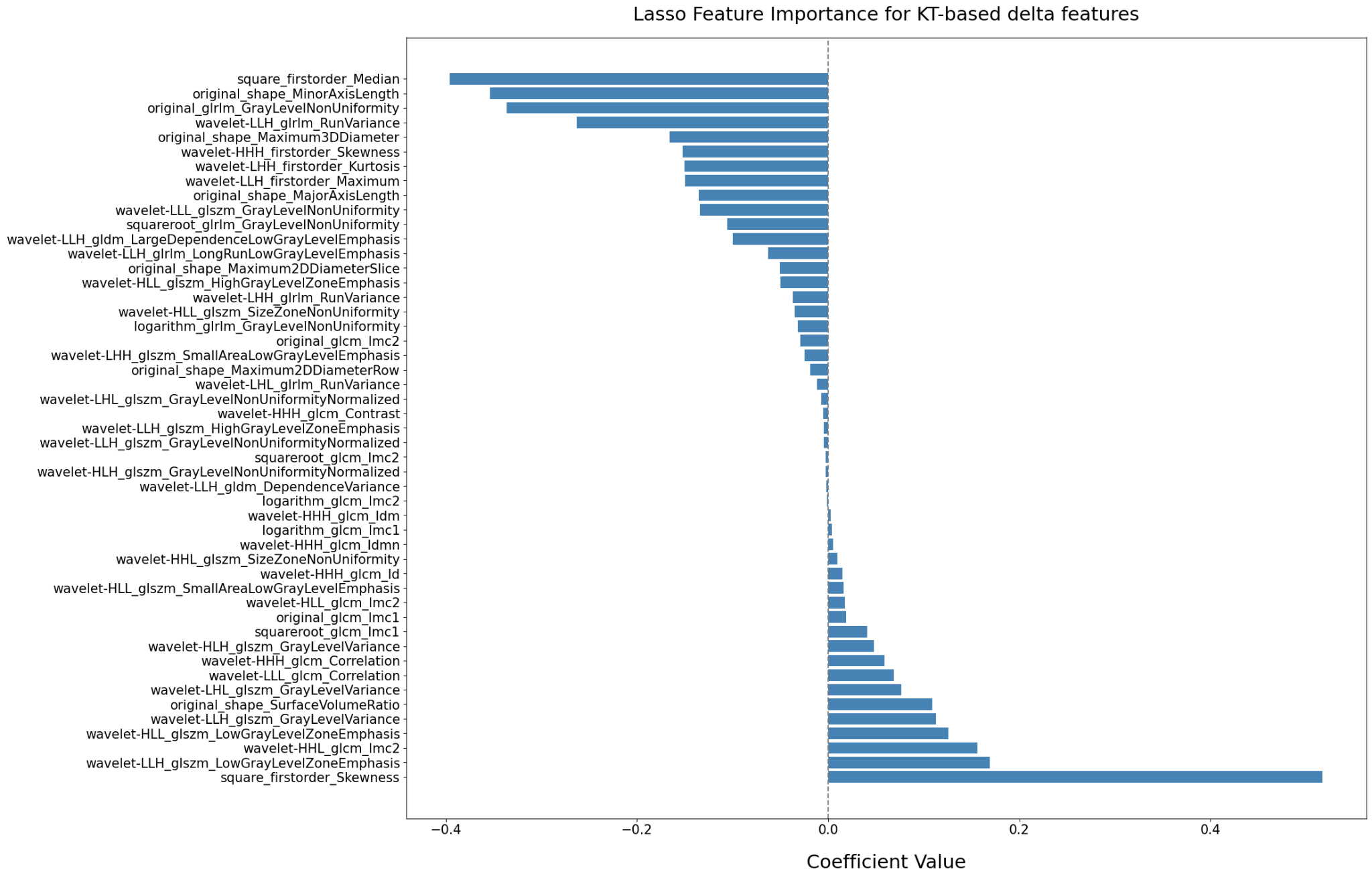


**(A)**


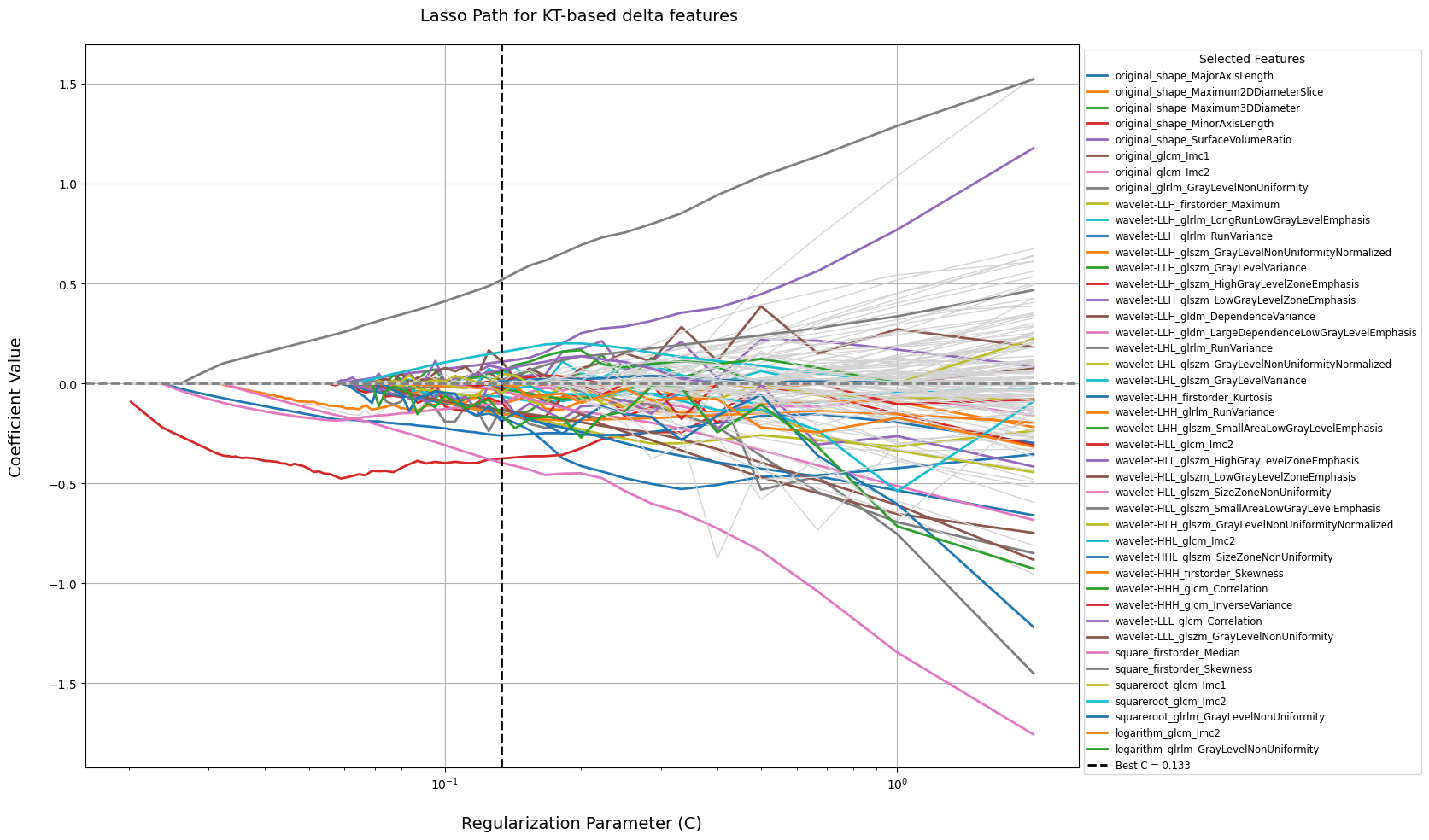


**(B)**

**Figure S6-** Feature importance (A) and Lasso curve (B) for 41 delta KT-based radiomic features selected by LASSO.


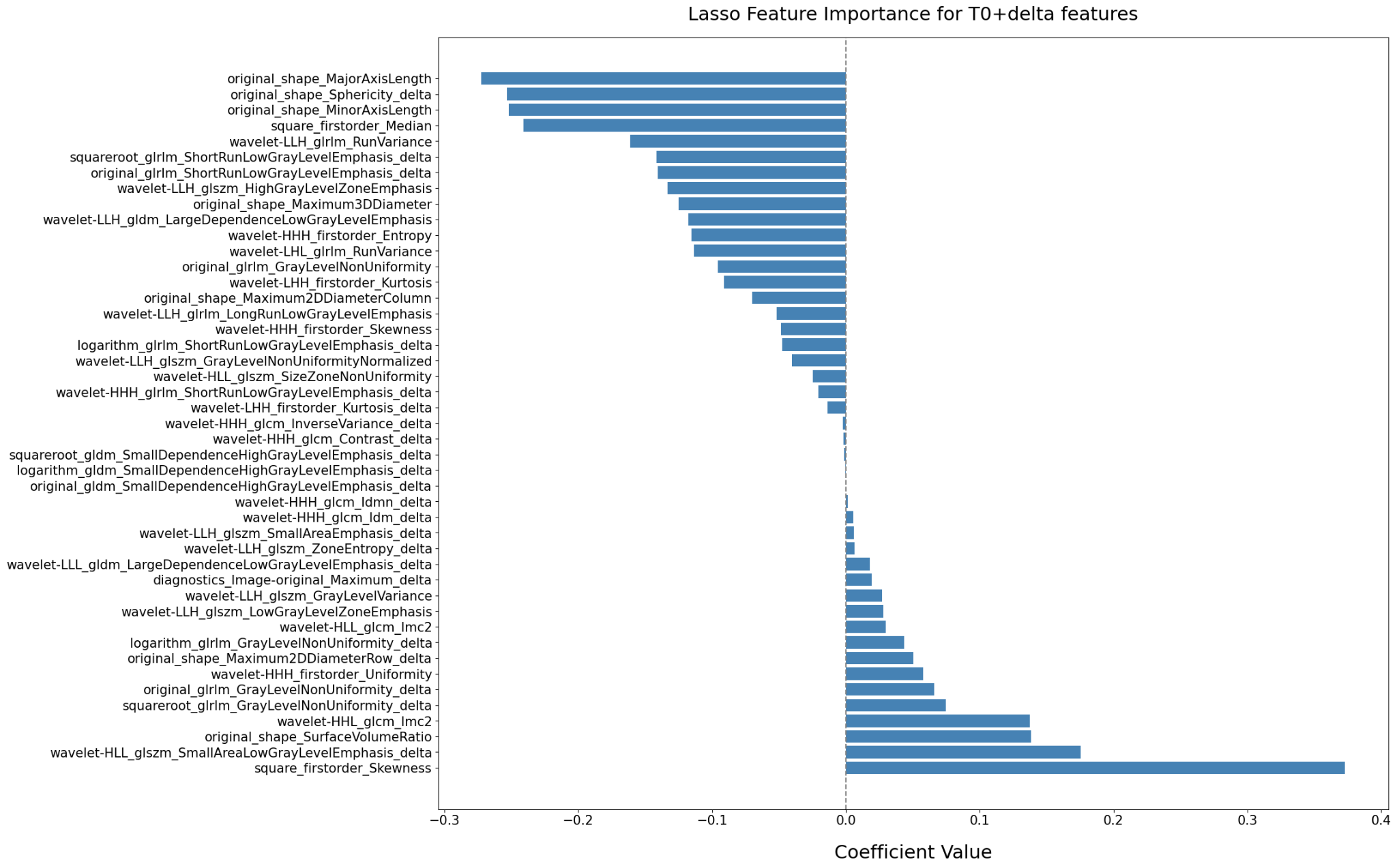


**(A)**


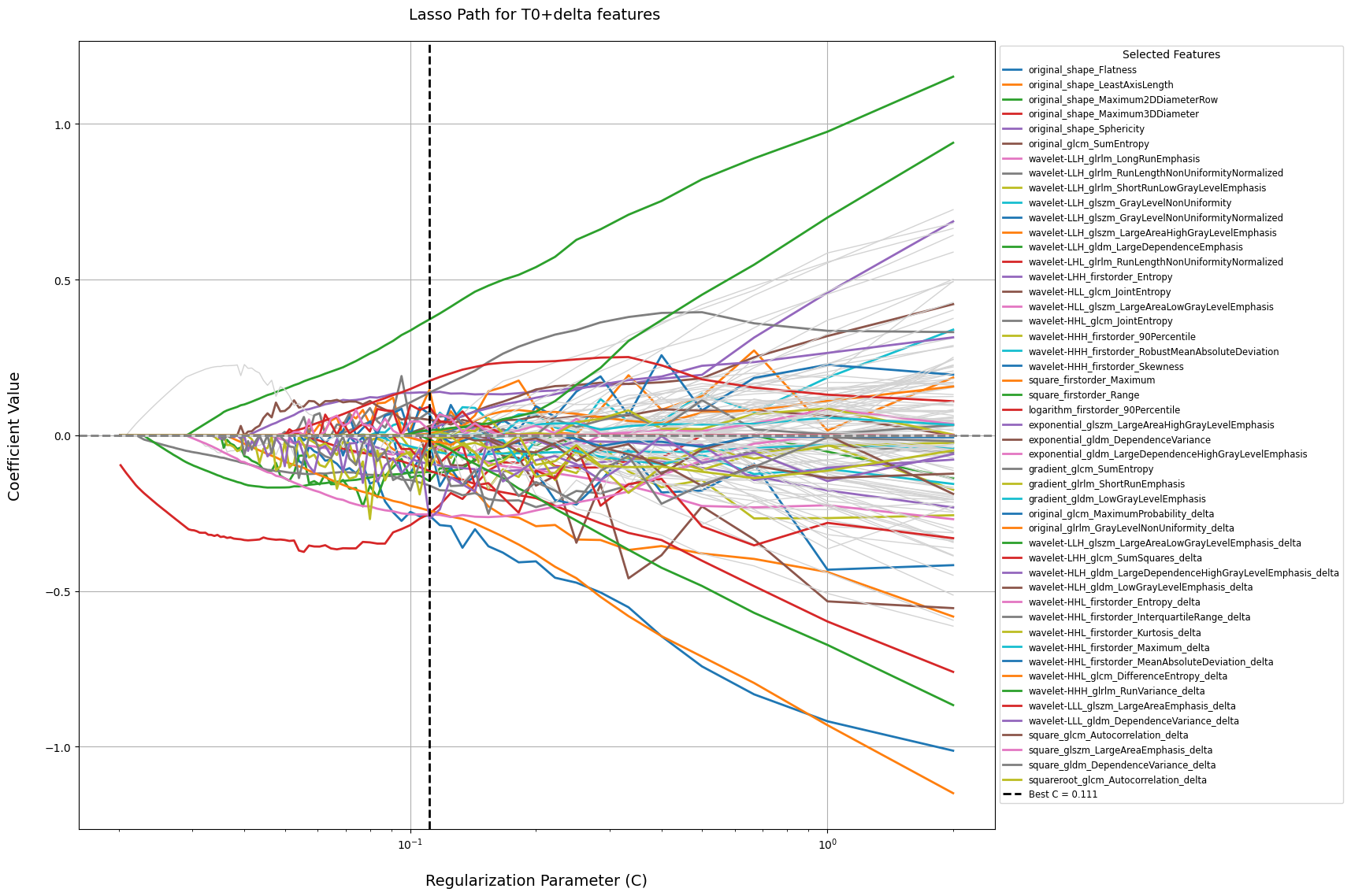


**(B)**

**Figure S7-** Feature importance (A) and Lasso curve (B) for 45 T0+delta not-harmonized radiomic features selected by LASSO.


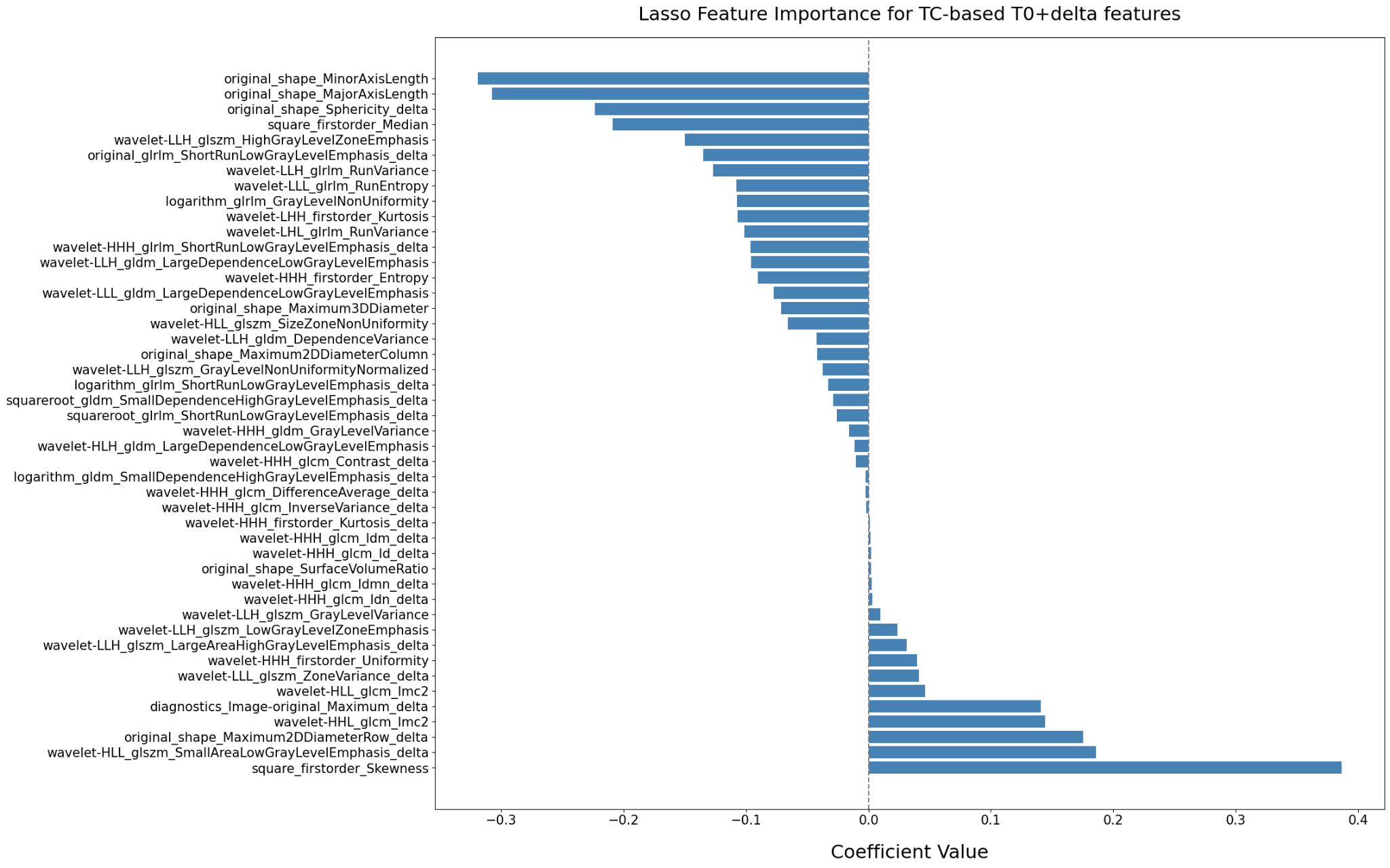


**(A)**


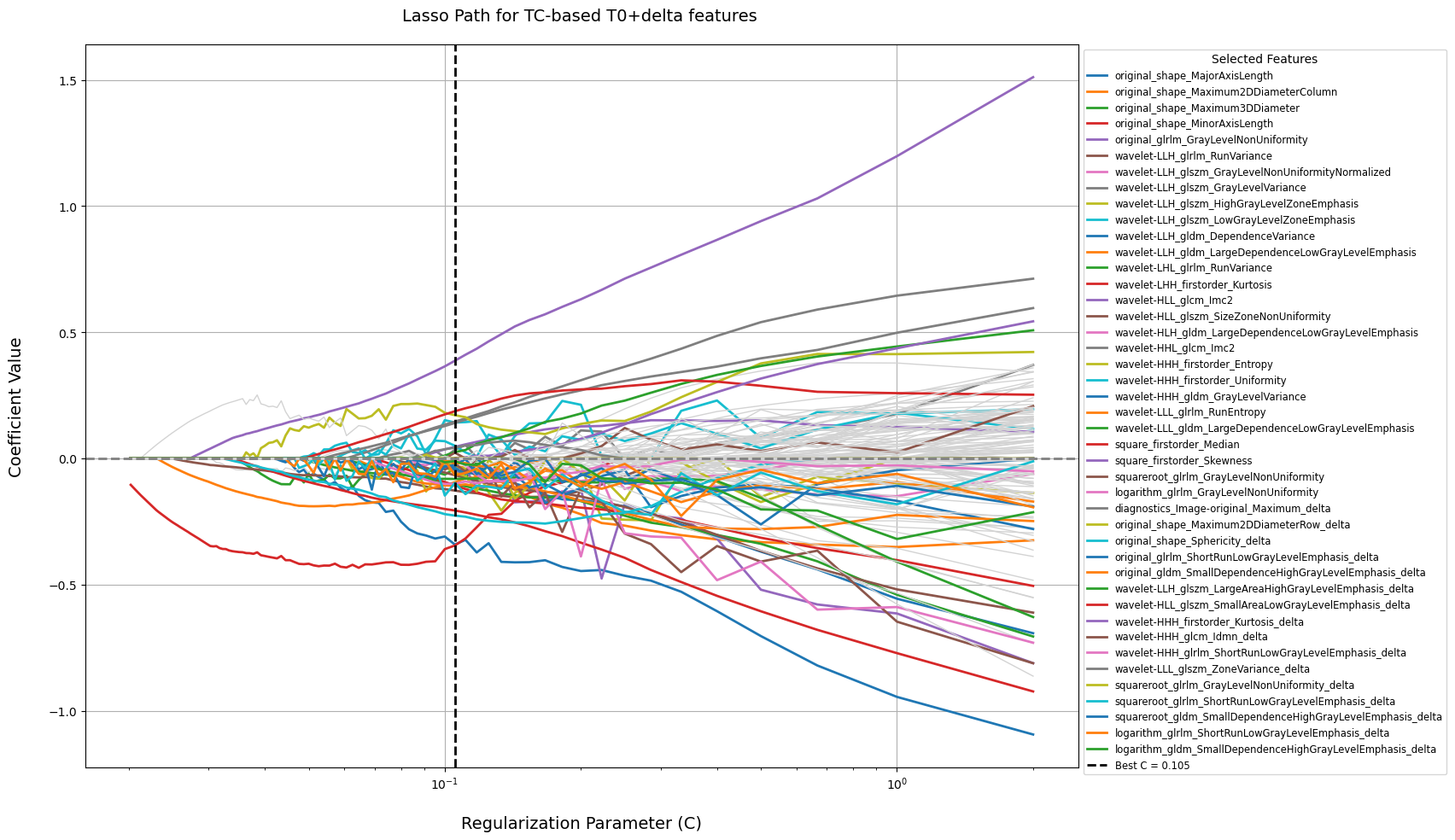


**(B)**

**Figure S8-** Feature importance (A) and Lasso curve (B) for 46 T0+delta TC-based radiomic features selected by LASSO.


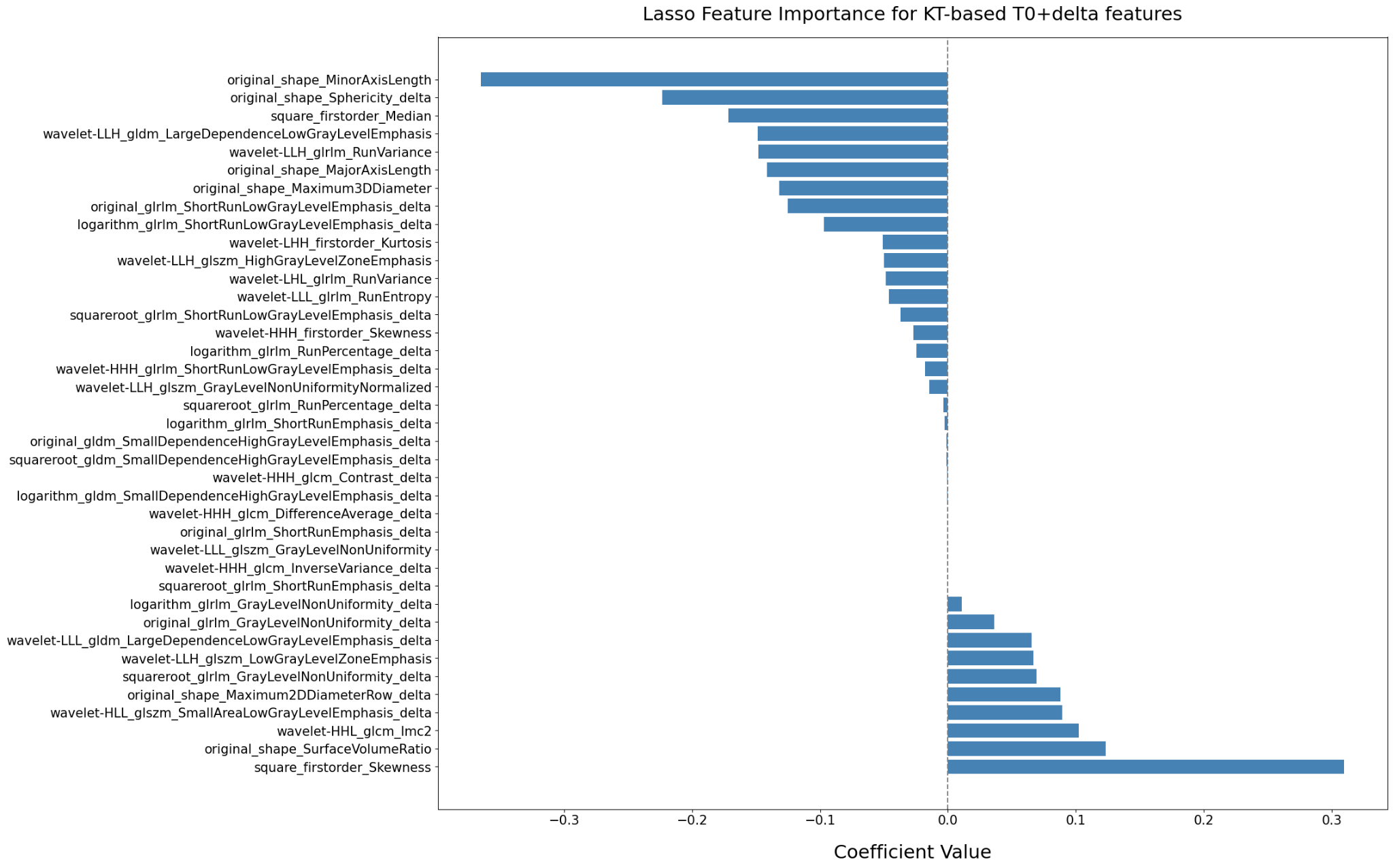


**(A)**


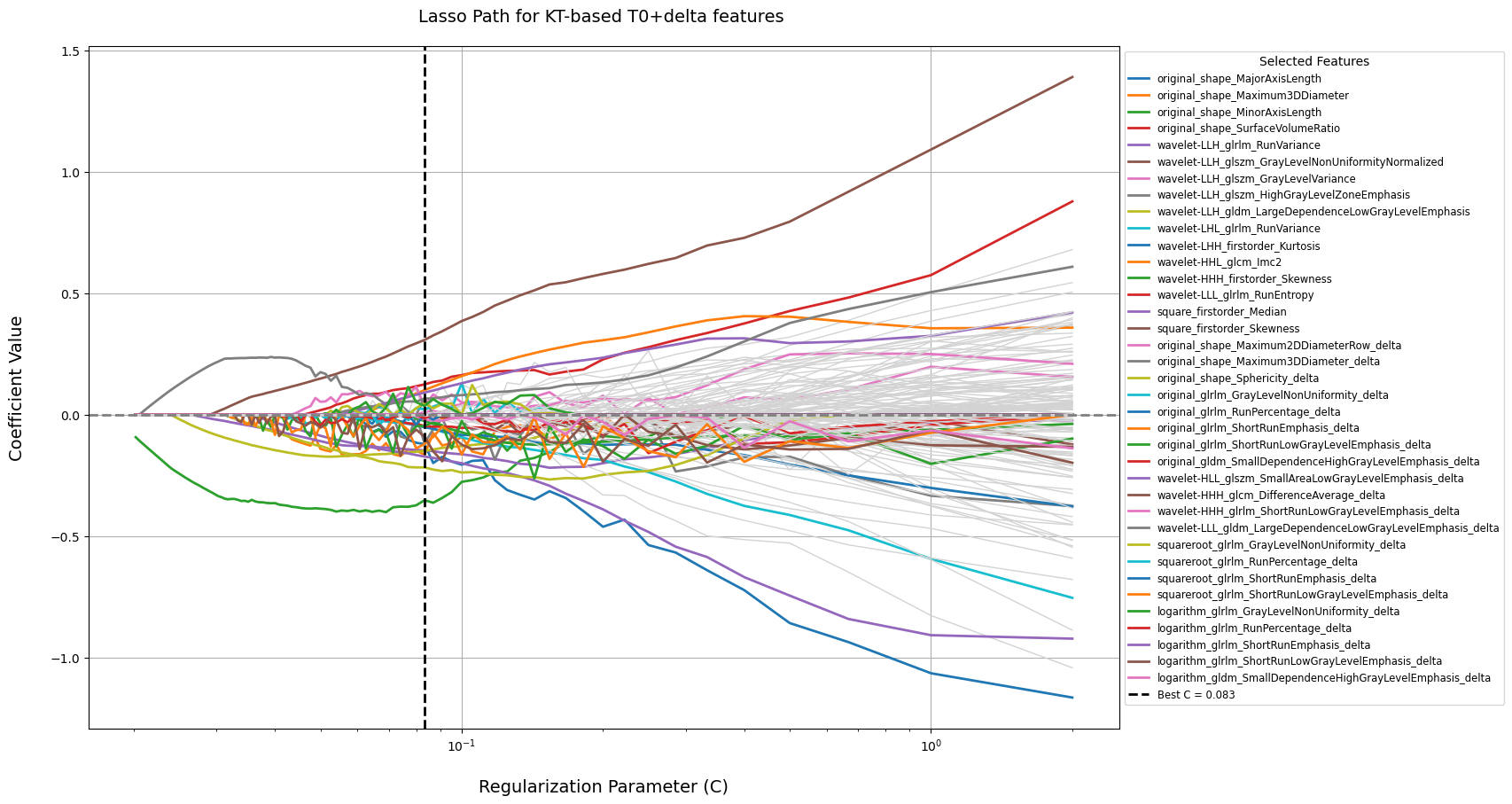


**(B)**

**Figure S9-** Feature importance (A) and Lasso curve (B) for 39 T0+delta KT-based radiomic features selected by LASSO.


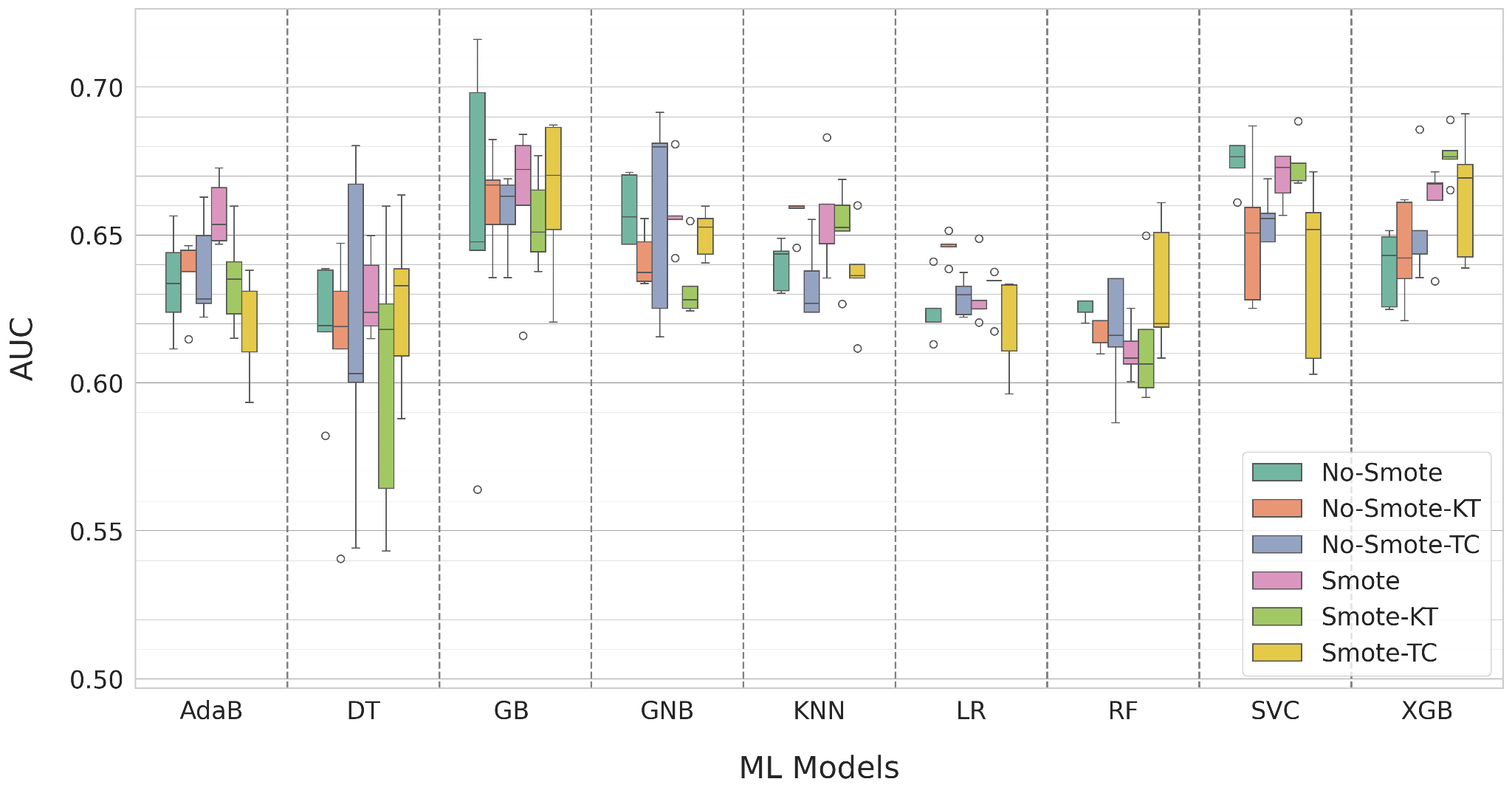


**(A)**

**
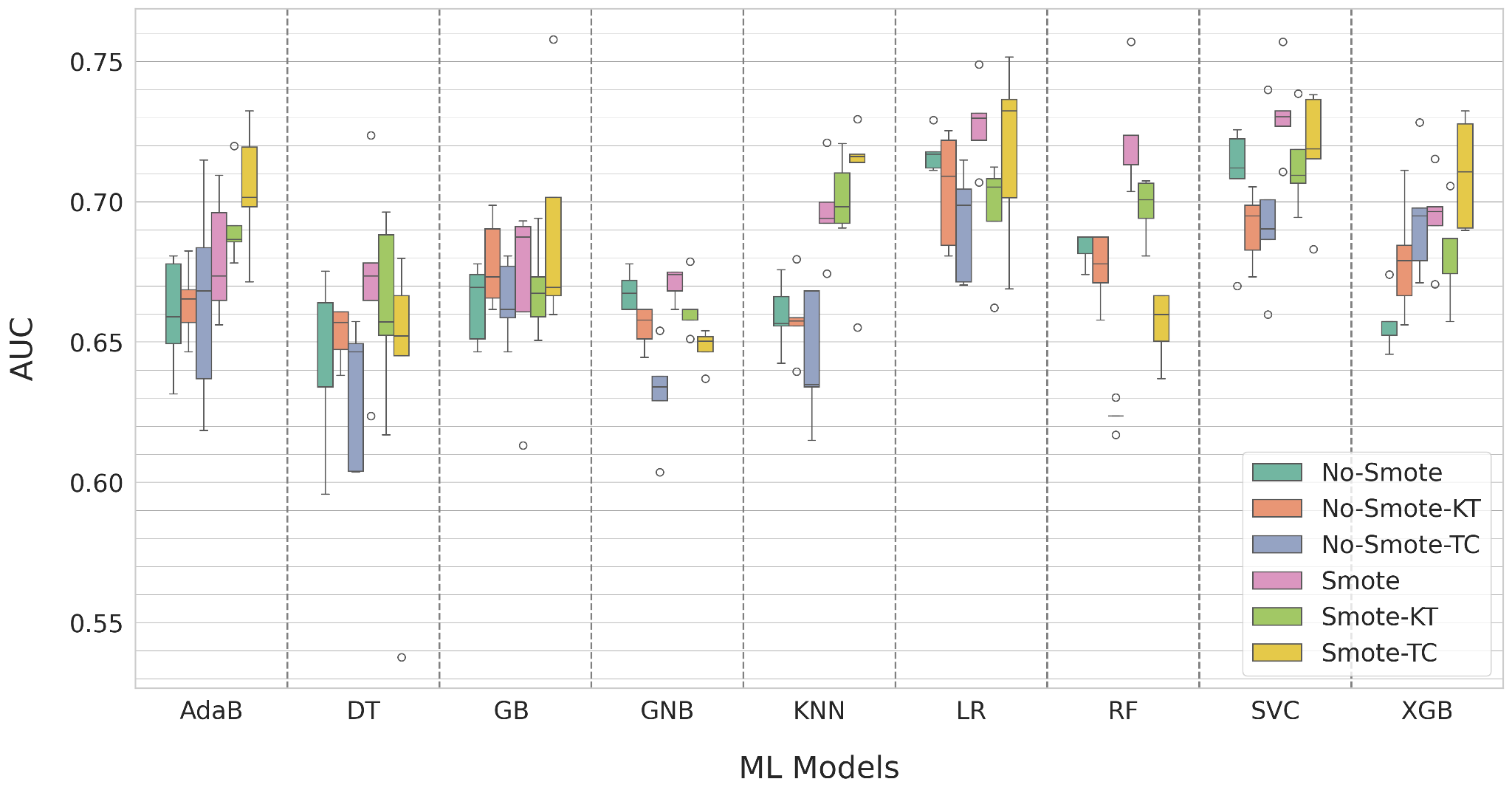
**

**(B)**

**
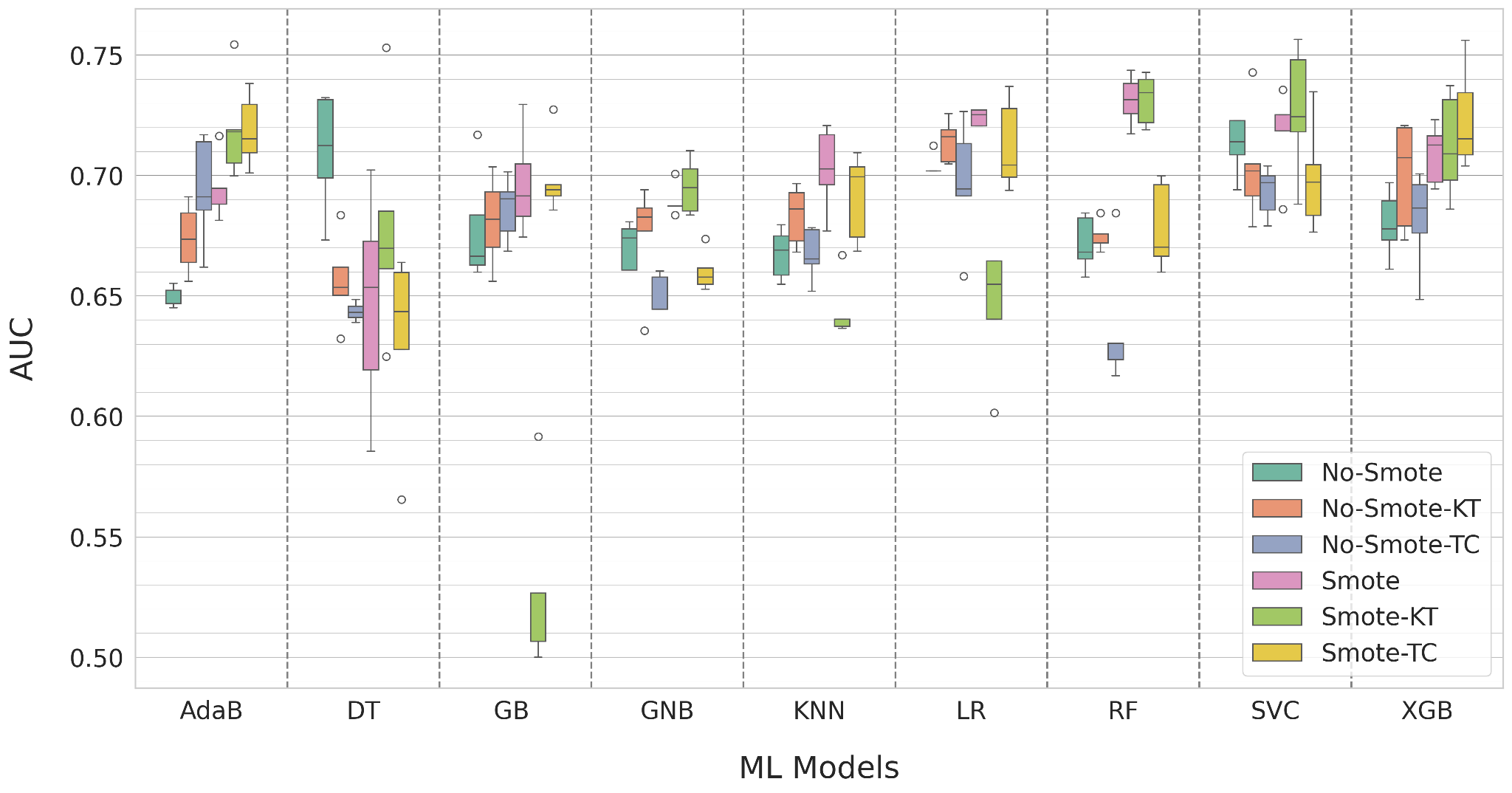
**

**(C)**

**Figure S10-** Median AUC of the ML models over feature selection methods using T0 (A), delta (B), and T0+delta (C) features.
